# An actionable GPCR restores phosphoinositide homeostasis and kidney function in Lowe syndrome

**DOI:** 10.1038/s41392-026-03054-z

**Published:** 2026-11-04

**Authors:** Leopoldo Staiano, Marialuisa Testa, Mariella Vicinanza, Francesca Cervellini, Laura Giaquinto, Lorenzo Patanella, Giuseppe Di Tullio, Michele Santoro, Annamaria Carissimo, Alessandro Giordano, Michele Genovese, Luis Juan Vicente Galietta, Anna Iervolino, Antonio Miele, Diego Luis Medina, Francesco Trepiccione, Francesco Emma, Olivier Devuyst, Robert Nussbaum, Maria Antonietta De Matteis

**Affiliations:** 1 Telethon Institute of Genetics and Medicine, Naples, Italy; 2 Institute for Genetic and Biomedical Research, National Research Council (IRGB-CNR), Milan, Italy; 3 Glaxo Smith Kline, Stevenage, UK; 4 Scuola Superiore Meridionale (School of Advanced Studies), Genomics and Experimental Medicine Program (GEM), Naples, Italy; 5 Department of Molecular Medicine and Medical Biotechnology (DMMBM), University of Naples “Federico II”, Naples, Italy; 6 Institute for Applied Mathematics “Mauro Picone”, National Research Council of Italy, Naples, Italy; 7 Department of Translational Medical Sciences (DISMET), University of Naples “Federico II”, Naples, Italy; 8 Biogem, Molecular Biology and Genetics Research Institute, Ariano Irpino, Italy; 9 Department of Translational Medical Sciences, University of Campania “L. Vanvitelli”, Naples, Italy; 10 Division of Nephrology, Ospedale Pediatrico Bambino Gesù IRCCS, Rome, Italy; 11 Institut de Recherche Experimentale et Clinique, UCLouvain, Brussels, Belgium; 12 Department of Physiology, University of Zurich, Zurich, Switzerland; 13 Labcorp (Formerly Invitae Corp), San Francisco, CA USA

**Keywords:** Kidney diseases, Cell biology, Drug screening, Medical genetics

## Abstract

Oculo-Cerebro-Renal-Lowe syndrome, caused by loss of the PI(4,5)P₂ 5-phosphatase OCRL, is a rare and severe disorder that lacks curative therapy. By integrating deep cell-biological insight into endosomal trafficking and proximal tubule protein reabsorption with high-content screening across engineered cell lines, patient-derived proximal tubule cells, human kidney organoids, and a humanized mouse model, we identify IB-MECA, an oral adenosine A3 receptor agonist, as a targeted therapy poised for clinical evaluation. IB-MECA normalizes endosomal PI(4,5)P₂ homeostasis, restores receptor trafficking, and rescues protein reabsorption in all systems, demonstrating the translational power of a mechanism-anchored, cross-species screening pipeline that couples cellular correction to organ-level benefit and supports rapid progression toward phase II testing pending regulatory approval. Mechanistic dissection revealed that IB-MECA acts through A3AR-dependent activation of PLC, revealing a previously unrecognized axis whereby receptor-driven hydrolysis of plasma-membrane PI(4,5)P₂ corrects pathological endosomal PI(4,5)P₂ accumulation. This receptor-mediated rewiring of phosphoinositide homeostasis contrasts with earlier strategies based on broad kinase inhibition and establishes plasma-membrane GPCR–PLC signaling as a precise, druggable entry point to correct endolysosomal lipid imbalance. Together with the favorable clinical safety record of IB-MECA, these findings provide a compelling rationale for an imminent clinical trial (NCT07410455) in Lowe syndrome and outline a generalizable, cell biology–guided framework for developing therapies for inherited disorders of phosphoinositide metabolism.

## Introduction

The precise spatial distribution of phosphoinositide species across the compartments of the endomembrane system is essential for proper cellular function. The critical importance of this organization is highlighted by the severe clinical consequences observed with loss-of-function mutations in genes encoding phosphoinositide-metabolizing enzymes.^1^ Such mutations lead to a wide spectrum of human disorders, most frequently stemming from defects in phosphoinositide phosphatases.^1^

A notable example is Oculo-Cerebro-Renal (Lowe) syndrome (OMIM #309000), a rare X-linked disorder caused by mutations in the OCRL gene,^2,3^ encoding a phosphatidylinositol 4,5-bisphosphate 5-phosphatase (PI(4,5)P₂ 5-phosphatase)^4,5^ that affects multiple organ systems, including the eyes, central nervous system, and kidneys.^6–9^ Ocular manifestations frequently include congenital bilateral cataracts and, in approximately 50% of cases, glaucoma.^7,9^ Neurological symptoms typically include severe neonatal hypotonia, variable severity of cognitive impairment, age-progressive behavioral abnormalities, and seizures in 30–50% of affected individuals.^7,9,10^ Renal involvement is characterized by proximal tubular dysfunction, manifesting as renal Fanconi syndrome. Fanconi syndrome manifests within the first months of life and shows marked variability in severity between individuals. Low–molecular-weight proteinuria and moderate albuminuria are consistently present, whereas glycosuria is only rarely detected. Additional renal findings include aminoaciduria and lysosomal enzymuria, which are almost invariably present, along with renal bicarbonate and phosphate wasting of variable degree.^6,11,12^ Hypercalciuria is a common finding, and nephrocalcinosis occurs in about half of affected individuals. In children, the glomerular filtration rate is typically mildly reduced (50–80 ml/min/1.73 m²) but often remains stable during the first decade of life, with a gradual decline thereafter.^6^ Indeed, the initial proximal tubule dysfunction often progresses via tubulointerstitial fibrosis to chronic kidney disease (CKD) and end-stage renal failure.^13^ Life expectancy is approximately 40 years on average, with mortality usually attributable to CKD-related complications.^7,9^ At present, no curative therapy is available.

Over the past two decades, investigations by several research groups, including our own, have demonstrated that loss of OCRL function—a 5-phosphatase that acts primarily on PI(4,5)P₂—results in the ectopic accumulation of PI(4,5)P₂ within the endolysosomal system, a compartment that normally contains this lipid at very low levels.^14,15^ This aberrant distribution impairs key trafficking pathways, particularly affecting the recycling of endocytic receptors from the early endosome to the plasma membrane (as with megalin/LRP2) or to the Golgi complex (as with the mannose-6-phosphate receptor, M6PR).^16,17^ Impaired recycling of these receptors from early endosomes is due to PI(4,5)P₂ accumulation, which induces excessive actin polymerization on endosomal membranes.^16^ In addition, PI(4,5)P₂ accumulation disrupts lysosomal function by interfering with transient reception potential mucolipin 1 (TRPML1)-mediated calcium signaling. The impaired TRPML1 function compromises both late endosome–lysosome fusion and autophagosome–lysosome fusion, thus hindering autophagic flux.^18^ In proximal tubule cells (PTCs), megalin entrapment inside early endosomes reduces its arrival to and its levels at the apical membrane. Megalin mis-trafficking underlies the defective protein reabsorption responsible for the low-molecular-weight proteinuria observed in Lowe syndrome (LS), while the impaired autophagic flux may further contribute to the progressive decline in renal function.^19,20^ Notably, these trafficking defects have been documented in a zebrafish model of LS,^21^ in the kidneys of *Ocrl*-deficient mice, as well as in PTCs and kidney biopsies from LS patients.^18,22^ These cellular abnormalities are closely and causally associated with an imbalance in PI(4,5)P₂ and can be rescued by chemically inhibiting or depleting phosphatidylinositol 4-kinases (PI4Ks) or phosphatidylinositol-4-phosphate 5-kinases (PIP5Ks), which are involved in PI(4,5)P₂ synthesis^,^^16,21^ or by modulating downstream effectors of ectopic PI(4,5)P₂ accumulation—specifically, actin dynamics and TRPML1 function in early endosomes and lysosomes, respectively.^18^ The tight link between these trafficking defects and LS pathogenesis, together with their demonstrated reversibility, highlights a valuable opportunity for therapeutic intervention. However, no approved drugs currently exist that selectively target the relevant 4- or 5-kinases.

Building on our access to a comprehensive set of LS models—including the well-characterized cellular and mouse systems as well as the LS human kidney organoids described here—we established a high-content screening platform to identify potential therapeutic compounds. Using a library of pharmacologically active molecules encompassing most approved drugs and candidates in advanced clinical trials, we leveraged our detailed understanding of LS cell biology to discover small molecules capable of restoring endocytic trafficking in both cellular and organoid contexts and reducing proteinuria in the LS mouse model. Through this screening approach, we identified IB-MECA, an adenosine A3 receptor (A3AR) agonist,^23^ as a lead compound that successfully advanced through all selection stages. Mechanistic studies revealed that IB-MECA acts via A3AR-dependent phospholipase C (PLC) activation, an effect shared by other PLC-coupled receptor agonists.^24–27^ These findings identify plasma membrane–derived PI(4,5)P₂ as a central driver of endolysosomal lipid imbalance in LS and highlight receptor-mediated signaling as a means to correct phosphoinositide dysregulation. Our results support the advancement of IB-MECA to clinical evaluation in LS, with the initiation of a clinical study (NCT07410455) now imminent.

## Results

### Primary screening identifies 113 active compounds that rescue the blockage of endosome-to-Golgi trafficking in OCRL-KD cells

The PI(4,5)P_2_ 5-phosphatase activity of OCRL is critical for regulating endocytic and M6PR trafficking through early endosomes (EEs) in PTCs from patients with LS and in established cell models of the disease.^16^ Previous work has demonstrated that reducing PIP5Kα levels decreases the level of PI(4,5)P_2_ and partially alleviates the defective endocytic trafficking characteristic of these cells.^16^ Additionally, PI(4,5)P_2_ levels can be decreased by inhibiting the formation of its precursor phosphatidylinositol 4-phosphate (PI4P); both genetic and chemical inhibition of PI4KIIIβ in OCRL-knockdown (KD) cells similarly rescued endocytic recycling, as measured by the redistribution of M6PR.^16,28^

**Fig. 1:**
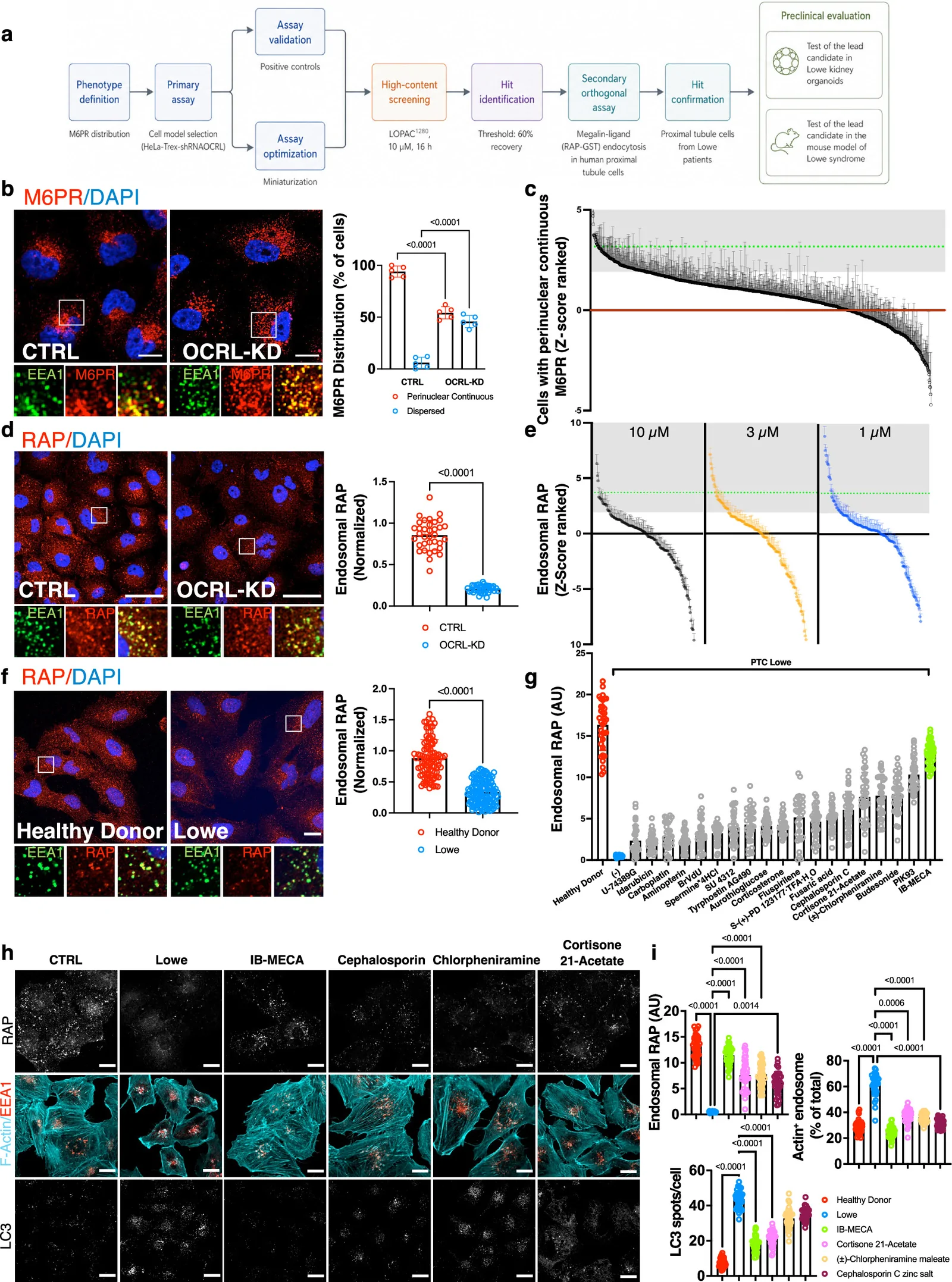
High-content screening of 1,280 pharmacologically active compounds revealed four hits that rescue endocytic and autophagic defects in PTCs from LS patients, and IB-MECA was prioritized for follow-up. **a** Pipeline of the high-content screen (HCS) applied for the identification of small molecules able to rescue the endocytic defects of cells lacking OCRL (generated with BioRender). **b** Representative images and quantitative analysis of M6PR distribution in control (CTRL) and OCRL-KD HeLa cells following tetracycline induction. M6PR is shown in red. The lower panels show magnifications of the boxed area, including EEA1 staining and the merged signal between M6PR and EEA1, to highlight the redistribution of M6PR relative to early endosomes. Nuclei were counterstained with DAPI (blue). [Scale bars, 20 µm]. **c** Primary HCS readout. LOPAC library compounds (1,280 total) were tested at 10 µM for 16 h in tetracycline-induced OCRL-KD HeLa cells. Compounds were ranked according to the Z score calculated from the percentage of cells displaying a perinuclear continuous M6PR pattern, with the OCRL-KD condition set to 0. Under this normalization, CTRL cells reached a Z score of 3.17, indicated by the green dashed line. A threshold corresponding to 60% rescue of the CTRL phenotype was set at a Z score of 1.9, indicated by the gray area, and was used to select compounds for further validation. Values are shown as empty circles and represent the mean Z score ± SD for each compound. **d** Representative images and quantitative analysis of RAP uptake, measured as the endosomal RAP (RAP/EEA1 fluorescence intensity), in control (CTRL) and OCRL-KD HK-2 cells exposed to RAP-GST for 5 minutes and stained for GST (red). The lower panels show magnifications of the boxed area, including EEA1 staining and the merged signal between RAP and EEA1, to highlight the endosomal localization of endocytosed RAP. Nuclei were counterstained with DAPI (blue). [Scale bars, 50 µm]. **e** Secondary screening. The 113 hits selected from the primary screening were retested in CTRL and OCRL-KD HK-2 cells at 10, 3, and 1 µM for 16 h. RAP uptake was measured as the endosomal RAP integrated density (RAP fluorescence intensity in endosomal area) per cell, and compounds were ranked according to the Z score calculated from the RAP uptake values, with the OCRL-KD condition set to 0. Under this normalization, CTRL cells reached a Z score of 3.7, indicated by the green dashed line. A threshold corresponding to 60% rescue of the CTRL phenotype was set at a Z score of 1.9, indicated by the gray area, and was used to select compounds for further validation. The three graphs show the Z scores for each compound at 10 µM, 3 µM, and 1 µM. The values are shown as filled circles and represent the mean Z score ± SD for each compound; black, orange, and blue circles indicate compounds tested at 10, 3, and 1 µM, respectively. **f** Representative images and quantitative analysis of RAP uptake, measured as the endosomal RAP (RAP/EEA1 fluorescence intensity ratio), in proximal tubule cells (PTCs) from a healthy donor and a Lowe patient exposed to RAP-GST for 5 minutes and stained for GST (red). The lower panels show magnifications of the boxed area, including EEA1 staining and the merged signal between RAP and EEA1, to highlight the endosomal localization of endocytosed RAP. Nuclei were counterstained with DAPI (blue). [Scale bars, 50 µm]. **g** Quantitative analysis of RAP uptake in PTCs from LS patients, measured as the endosomal RAP integrated density (RAP fluorescence intensity in the endosomal area) per cell, after treatment with 18 compounds (10 μM) selected from the 113 tested in (**e**). PIK-93 (500 nM) was included as a positive control. IB-MECA treatment is highlighted in green. **h** Representative images of the top four performers across assays. The four compounds were tested in healthy donor (CTRL) and Lowe PTCs for 16 h at 10 µM. Cells were tested for RAP uptake (white) (as in **f**) or immunolabeled with anti-EEA1 (red) and phalloidin (cyan) to label early endosomes and F-actin, respectively, or with anti-LC3 antibody (white) to label autophagosomes. [Scale bars, 20 µm]. **i** Quantitative analysis of the conditions in (**h**). For the graphs in (**b**), (**g**), and (**i**), each spot  represents average value calculated per individual cells from fields including at least 20 cells ; for the graphs in (**d**), (**f**), each spot represents a cell. For **b**, **g**, **i**, the graph is based on measurements from at least 100 cells per condition across three independent biological replicates (total *n* > 300, *N* = 3). For **d**, **f**, the graph is based on measurements from at least 50 cells per condition across three independent biological replicates (total *n* > 150, *N* = 3). The Kruskal–Wallis test was used for statistical analysis, and *p* values < 0.05 are reported on the graph

To identify possible pharmacological correctors for LS, a high-content screening (HCS) pipeline was developed on the basis of a phenotypic, cell-based approach (Fig. 1a). A stable, tetracycline-inducible OCRL-KD HeLa cell line was generated (see Methods). Following tetracycline induction, OCRL protein levels were reduced by approximately 80% (Supplementary Fig. 1a). This knockdown resulted in the loss of the typical perinuclear/Golgi localization of M6PR, which redistributed into peripheral EEA1-positive structures as previously observed^16^ (Fig. 1b). Notably, the PI4KIIIβ inhibitor PIK93^28,29^ robustly restored perinuclear M6PR localization in these OCRL-deficient cells and served as a positive control for screening (Supplementary Fig. 1b).

The screening assay was based on this altered M6PR pattern in OCRL-KD cells. The Library of Pharmacologically Active Compounds (LOPAC; Sigma Aldrich), containing 1,280 small molecules, including FDA-approved drugs and compounds at advanced stages of clinical development, was tested. Compounds were applied at a fixed concentration of 10 μM for 16 hours to tetracycline-induced OCRL-KD cells, which were then fixed, stained for M6PR, and counterstained with DAPI. Among the initial 1280 compounds, 113 were found to partially or fully correct retrograde M6PR trafficking from EEs to the Golgi. Compounds that restored perinuclear M6PR localization in more than 60% of the OCRL-KD cells were classified as positive hits (Fig. 1c, Supplementary Table 1).

### Secondary and validation assays establish IB-MECA as the top-performing corrector in LS cell models

The 113 active compounds from the primary screen were subsequently evaluated in a secondary assay using a human proximal tubule (HK-2) cell line, focusing on endocytic uptake of megalin ligands, a process directly linked to renal dysfunction in LS.^16^ For this purpose, the uptake of receptor-associated protein (RAP), a chaperone that binds megalin, was used as a well-established marker for megalin-mediated endocytosis.^30^ A RAP uptake assay, in which OCRL-KD cells were previously shown to be defective^16^ (Fig. 1d), was adapted to a 384-well format (see Methods) and was used to assess compound efficacy at three concentrations (1, 3, and 10 μM) in both control and OCRL-KD HK-2 cells (Fig. 1e). Eighteen compounds were able, at two or more concentrations, to restore RAP uptake to at least 50% of control levels (Supplementary Fig. 1c).

For validation, the ability of these 18 compounds to rescue endocytic defects was tested in PTCs derived from LS patients (Fig. 1f, g) and healthy donors (Supplementary Fig. 1d). In addition to RAP uptake, other phenotypes related to the loss of function of OCRL were evaluated, including actin stress fiber disruption with endosomal actin accumulation and increased autophagosome numbers in both healthy donor and Lowe patient-derived PTCs^16,18^ (Supplementary Fig. 1e, f). The top four compounds identified across all the assays (Fig. 1h, i) were cortisone 21-acetate (a prodrug with both glucocorticoid and mineralocorticoid activity), chlorpheniramine (an antihistamine), cephalosporin (a β-lactam antibiotic), and IB-MECA (an A3 adenosine receptor agonist).

Among these compounds, IB-MECA (also known as Piclidenoson or CF-101), a first-in-class A3 receptor (A3AR) agonist, showed superior efficacy in correcting endocytic and actin defects in patient-derived cells. Although IB-MECA is not commercially available, it has been evaluated in both preclinical^31–35^ and clinical settings, including a successful phase III trial, where it demonstrated significant anti-psoriatic activity and a highly favorable safety profile.^36,37^ On the basis of these findings, IB-MECA was prioritized for further validation and for in vivo studies in models of LS.

### IB-MECA restores the protein reabsorption capacity of OCRL-KO kidney organoids

The data presented above consistently demonstrate the robust corrective effect of IB-MECA on endocytic defects resulting from OCRL loss in 2D cell culture. To evaluate IB-MECA in a more physiologically relevant system, we generated kidney organoids from wild-type (WT) and OCRL-knockout (OCRL-KO) induced pluripotent stem cells (iPSCs).^38,39^

**Fig. 2:**
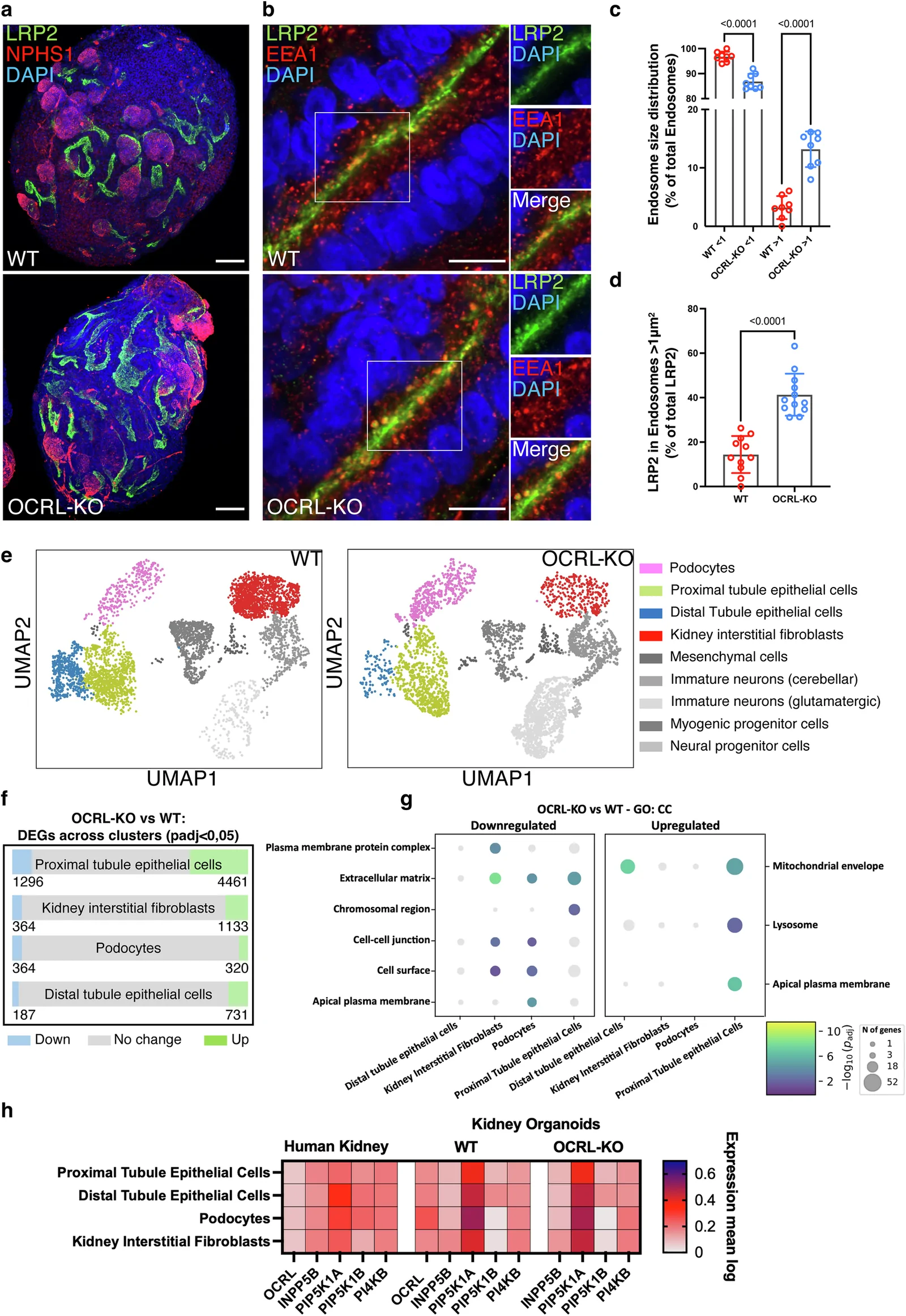
OCRL-KO kidney organoids maintain nephron morphology and lineage composition, with selective endosomal remodeling and lysosomal/mitochondrial gene upregulation in proximal tubules. **a** Kidney organoids generated from WT (isogenic control) and OCRL-KO iPS cells display anatomically ordered nephron segments. Glomerular cells are labeled for Nephrin/NPHS1 (red), and proximal tubules are labeled for LRP2/Megalin (green). The nuclei were counterstained with DAPI. [Scale bars, 100 µm]. **b** Representative images of WT and OCRL-KO proximal tubules in kidney organoids labeled for LRP2 (green) and EEA1 (red). [Scale bars, 10 µm]. **c** Quantitative analysis of the percentage of small (area <1 µm²) and large EEA1⁺ early endosomes (area > 1 µm²) in proximal tubule cells from WT (red) and OCRL-KO (blue) organoids. Each dot represents the percentage of small ( < 1 µm²) and large ( > 1 µm²) EEA1⁺ endosomes in a single organoid (*n* = 8 organoids from 3 independent experiments). Statistical significance was assessed using a Kruskal–Wallis test; the corresponding *p* value is indicated on the graph. **d** Quantitative analysis of the fluorescence intensity of LRP2 in large endosomes in the proximal tubules of WT (red) and OCRL-KO (blue) organoids. For each organoid, the total LRP2 fluorescence per proximal tubule and the LRP2 fluorescence localized to large EEA1⁺ endosomes (area > 1 µm²) were measured, and the percentage of endosomal LRP2 relative to total LRP2 was calculated. Each dot represents the mean value per organoid (*n* = 12 organoids from 3 independent experiments). The Kruskal‒Wallis test was used for statistical analysis, and the *p* values are reported on the graph. **e** Single-cell RNA-seq UMAPs show comparable cell-type compositions between WT and OCRL-KO organoids and no detectable lineage imbalance. UMAP embeddings of integrated scRNA-seq data from WT and OCRL-KO organoids show the annotated cell populations (distal tubule epithelial cells, proximal tubule epithelial cells, podocytes, kidney interstitial fibroblasts, and additional minor/immature clusters, as indicated in the legend). **f** Differential gene expression summary (OCRL-KO vs WT; adjusted *p* < 0.05) across major kidney clusters. The full data are provided in Supplementary Data 1. The bars indicate the number of genes whose expression was downregulated (blue), unchanged (gray), or upregulated (green) in OCRL-KO organoids compared with WT organoids. **g** Gene Ontology (GO) cellular component (CC) enrichment for genes whose expression was downregulated and upregulated in OCRL-KO vs WT mice, as shown per cluster. Dot size represents the number of genes contributing to each term; dot color indicates enrichment significance ( − log10 adjusted *p*). The GO categories shown were selected from the complete enrichment analysis (Supplementary Data 2) on the basis of the filtering criteria (shown in Supplementary Data 2) intended to pinpoint nonredundant and interpretable GO:CC categories. **h** Heatmap showing normalized mean expression levels of selected enzymes involved in PI(4)P and PI(4,5)P₂ metabolism across matched cell populations in human kidney and human kidney organoids. The analyzed genes included OCRL, INPP5B, PIP5K1A, PIP5K1B, and PI4KB. The same cell populations were considered in the human kidney dataset and in WT and OCRL-KO kidney organoids to allow direct comparison of their expression profiles. Human kidney data were derived from the publicly available single-cell RNA-seq dataset GSE234788, while organoid data were obtained from single-cell RNA-seq of the WT and OCRL-KO kidney organoids generated in this study. The normalized mean expression is shown for each gene and cell type; expression values were calculated from the raw counts by normalizing each gene count to the total per-cell library size and applying a log1p transformation: log1p(raw count/total counts per cell × 10,000)

By optimizing a published protocol,^40,41^ we successfully produced, within a few weeks, hundreds of homogeneous kidney organoids suitable for reproducible treatments and assays. Differentiation efficiency was confirmed by the expression of nephron-segment–specific genes (Supplementary Fig. 2a) and by the presence of anatomically organized nephron structures, ranging from the glomerulus to the proximal tubule, with glomerular cells labeled by Nephrin (NPHS1) and proximal tubules (PTs) marked by LRP2/Megalin (Fig. 2a). Morphologically, the WT and OCRL-KO kidney organoids were indistinguishable on day 21. The only notable differences were the presence of enlarged EEs and prominent endosomal LRP2/megalin accumulation in OCRL-KO PTs compared with those in WT PTs, findings that confirm and validate previous observations in 2D kidney cell culture (Fig. 2b–d).

Single-cell RNA sequencing analysis revealed no overt developmental defects or major lineage imbalances. WT and OCRL-KO organoids displayed broadly comparable cell-type compositions, and all principal nephron cell classes were robustly identified, together with the expected extrarenal cell types (immune and neural cells), under all conditions (Fig. 2e).

Analysis of the impact of OCRL knockout on gene expression profiles revealed that PT cells were the most strongly affected. In these cells, 4,461 genes were upregulated, and 1,296 genes were downregulated, a magnitude far greater than that seen in other kidney-specific clusters, including podocytes (320 up, 364 down), distal nephron epithelial cells (731 up, 187 down), and interstitial fibroblasts (1,133 up, 364 down; Fig. 2f and Supplementary Data 1). These findings highlight the central role of OCRL in PT cells and provide a compelling explanation for their pronounced vulnerability to OCRL loss of function, which underlies the renal Fanconi syndrome characteristic of LS.

Gene set enrichment analysis (GSEA) of OCRL-KO-regulated genes in PT cells was performed using GO cellular component (GO:CC) terms, and the results further refined this picture (Fig. 2g and Supplementary Data 2). GO:CC terms containing more than 1000 or fewer than 100 genes were excluded, and the most representative terms were selected from the remaining categories to minimize redundancy among GO classes sharing largely overlapping gene sets. The upregulated genes were strongly enriched in categories related to the mitochondrial envelope, lysosomes, and the apical plasma membrane. This pattern mirrors our earlier transcriptomic results in PT cell lines,^18^ where OCRL loss induced TFEB-dependent upregulation of mitochondrial and lysosomal genes (Fig. 2g).

The enrichment of apical plasma membrane–associated genes likely reflects an adaptive response to defective apical endocytic recycling, driving increased expression of membrane-targeted proteins—including endocytic components and transporters—to compensate for reduced uptake. Consistent with this, the selective induction of key apical endocytic, recycling, and transporter modules in PT cells suggests the activation of a compensation program aimed at strengthening apical surface function and mitigating OCRL-dependent trafficking defects (Supplementary Data 1, 2). Conversely, downregulated genes in PT cells were enriched for terms associated with chromatin organization and the extracellular matrix (Fig. 2g and Supplementary Data 1, 2), whose functional implications remain to be clarified.

We also performed a focused analysis of the expression of PI4Ks and PIP5Ks (PI4KB, PIP5K1A and PIP5K1B), which generate the PI(4,5)P₂ pool regulated by OCRL,^16,21^ as well as INPP5B, the 5‑phosphatase whose high expression levels compensate for OCRL loss in mice^42,43^ (Fig. 2h). We compared the expression levels of these genes in human kidney organoids with available transcriptomic data from human kidneys^44^ and observed a similar relative abundance. Notably, these levels remained comparable in the OCRL‑KO and WT organoids, indicating that OCRL loss is not associated with transcriptional rewiring of the core PI‑metabolizing machinery.

**Fig. 3:**
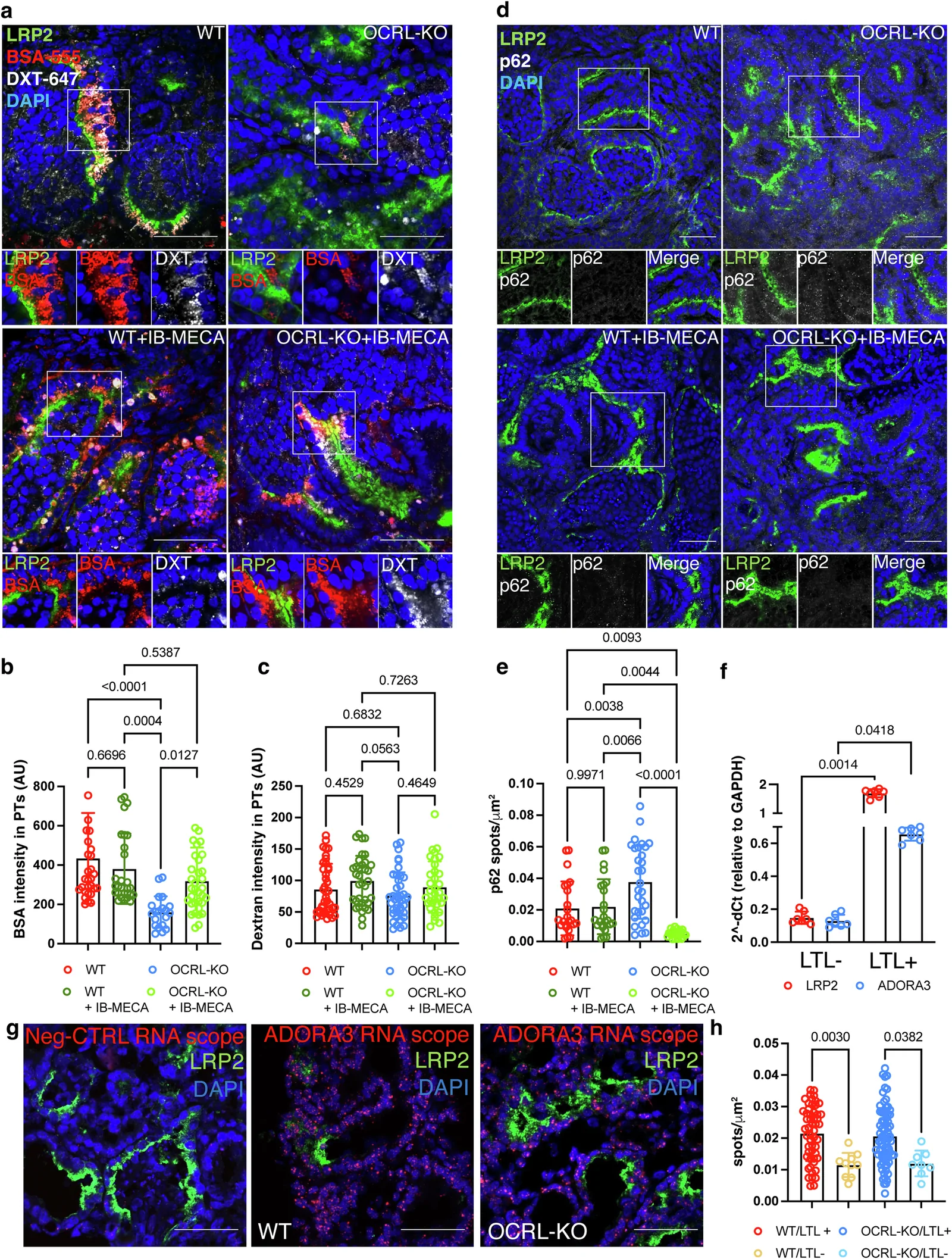
IB-MECA restores receptor-mediated endocytosis and autophagic flux in OCRL-KO kidney organoids. Representative images (**a**) and results of the quantitative analysis of proximal-tubule receptor-mediated endocytosis (**b**) and fluid-phase endocytosis (**c**) by fluorescent albumin (BSA-568, red) and dextran (DXT-647, white), respectively, within LRP2+ (green) tubules in WT and OCRL-KO kidney organoids either untreated or treated for 7 days (from day 14 to day 21) with IB-MECA (10 μM). Boxed regions in the images are shown at higher magnification in the lower panels. Each point represents the BSA-555 or DXT-647 fluorescence intensity in a single proximal tubule. The nuclei were counterstained with DAPI. [Scale bars, 50 µm]. **d** Representative images and **e** quantitative analysis of autophagy/lysosomal flux measured as the number of spots of the autophagy cargo receptor p62 (white) within LRP2+ (green) proximal tubules in WT and OCRL-KO kidney organoids either left untreated or treated for 7 days (from day 14 to day 21) with IB-MECA (10 μM). The number of p62-positive spots normalized to the proximal tubule area (p62 spots/µm²) is reported. [Scale bars, 50 µm]. For **b**, **c**, **e**, statistical analysis was performed by one-way ANOVA with Tukey’s post hoc test, and *p* values are reported on the graph (data are from *n* > 30 proximal tubules ( > 4 tubules per organoid) from *N* = 9 organoids derived from 3 independent differentiations (3 organoids per differentiation). **f** qPCR analysis of LRP2 and ADORA3 expression in LTL+ proximal tubule cells and LTL− cells isolated from human kidney organoids by FACS sorting. LRP2 was used to validate the enrichment of proximal tubule cells in the LTL+ fraction, while ADORA3 expression was measured to assess its expression in proximal tubule cells compared with that in the LTL− population. Data were obtained from three independent biological replicates, each generated by sorting cells from 70 kidney organoids. The values are shown as the mean ± SD. **g** RNAscope analysis of ADORA3 mRNA expression in WT and OCRL-KO human kidney organoid cryosections. ADORA3 transcripts are shown in red, proximal tubules were identified by LRP2 staining in green, and nuclei were counterstained with DAPI. A negative-control RNAscope probe is shown to assess the background signal. [Scale bars, 50 µm]. **h** Quantitative analysis of the data in **g** shows the number of ADORA3 RNAscope puncta normalized to the area (µm²) in LRP2-positive proximal tubule regions and extratubular regions of WT and OCRL-KO kidney organoids. Data were obtained from fifteen organoids across three independent biological replicates (5 organoids per replicate). The values are shown as the mean ± SD. Statistical analysis was performed using one-way ANOVA followed by Tukey’s post hoc test

To evaluate organoid functionality, we developed endocytic assays measuring both fluid-phase (dextran uptake) and receptor-mediated (albumin uptake) endocytosis. WT and OCRL-KO organoids internalized dextran to a similar extent (Fig. 3a, c). However, while the WT organoids efficiently internalized fluorescent bovine serum albumin (BSA-555) within the LRP2+ PTs, the OCRL-KO organoids displayed a marked ( > 2-fold) reduction in albumin uptake (Fig. 3a, b).

These findings, which are consistent with the results from the LS cell models, prompted us to test whether IB-MECA could rescue the endocytic defects in the OCRL-KO kidney organoids.

Treatment with IB-MECA (10 μM for 7 days, from day 14 to day 21) significantly increased albumin uptake in the OCRL-KO organoids and restored the endocytic function of the proximal tubules to WT levels but did not affect albumin uptake in the WT organoids (Fig. 3a, b). This rescue was mechanistically specific: fluid-phase dextran uptake (Fig. 3c), which, as noted above, was not perturbed by OCRL deficiency, was unaffected by IB-MECA, indicating that IB-MECA specifically targets the receptor-mediated pathway.

In addition to impaired receptor-mediated albumin uptake, OCRL-KO kidney organoids—consistent with data from cell and animal models and human kidney biopsies—exhibited defective autophagic flux, as evidenced by increased p62/SQSTM1 puncta within the proximal tubules. IB-MECA treatment, which had no effect on basal p62/SQSTM1 levels in WT organoids, markedly reduced the number of p62/SQSTM1 puncta (Fig. 3d, e), confirming the ability of IB-MECA to restore deficient autophagic flux induced by OCRL loss of function. The expression of the IB-MECA target receptor A3AR was validated in kidney organoids, particularly in proximal tubular cells, by orthogonal approaches, including RT-qPCR (Fig. 3f), RNAscope (Fig. 3g, h) and immunofluorescence (Supplementary Fig. 3a, b). Consistent with these findings, ADORA3 expression was also detected in PTCs from healthy donors and Lowe syndrome patients (Supplementary Fig. 3c, d) and in HK-2 cells (Supplementary Fig. 3e, f).

Importantly, consistent with the favorable safety profile of IB-MECA observed in both preclinical and clinical studies, treatment with IB-MECA did not have a major effect on organoid differentiation. Indeed, the Uniform Manifold Approximation and Projection (UMAP) profiles of the WT and OCRL-KO organoids completely overlap, irrespective of treatment status (Supplementary Fig. 4a). In general, the global gene- expression profile of OCRL-KO kidney organoids was not markedly affected by IB-MECA treatment (Supplementary Data 1, 3). Only a subset of genes whose expression was deregulated in the OCRL-KO organoids were partially normalized upon IB-MECA exposure (Supplementary Fig. 4b; Supplementary Data 1, 3). Intriguingly, IB-MECA administration led to the significant upregulation of the expression of MCOLN1, which encodes the transient receptor potential mucolipin 1 (TRPML1) channel (Supplementary Data 1, 3), whose impaired function is known to disrupt autophagic flux in OCRL-KO cells. Building on our previous findings that increased MCOLN1/TRPML1 expression and activity can restore autophagic flux in OCRL-depleted cells, we propose that IB-MECA–induced MCOLN1/TRPML1 upregulation may contribute, at least in part, to the recovery of autophagy observed in OCRL-KO kidney organoids. Importantly, IB-MECA treatment did not alter the expression of the main PI-metabolizing enzymes (Supplementary Data 1), ruling out the possibility that correction of the endocytic defects in OCRL-KO organoids reflects a transcriptional induction of compensatory phosphoinositide-metabolizing enzymes.

### IB-MECA rescues proteinuria in a mouse model of Lowe syndrome

After confirming that IB-MECA can restore protein resorption in OCRL-KO kidney organoids, we sought to assess its effects at the organismal level using a humanized *Ocrl*^*Y/-*^ mouse model of LS.^45^ This model recapitulates key renal features of LS, including significant urinary loss of low-molecular-weight (LMW) proteins and albumin due to impaired receptor-mediated endocytosis in proximal tubule cells.^22,45^

**Fig. 4:**
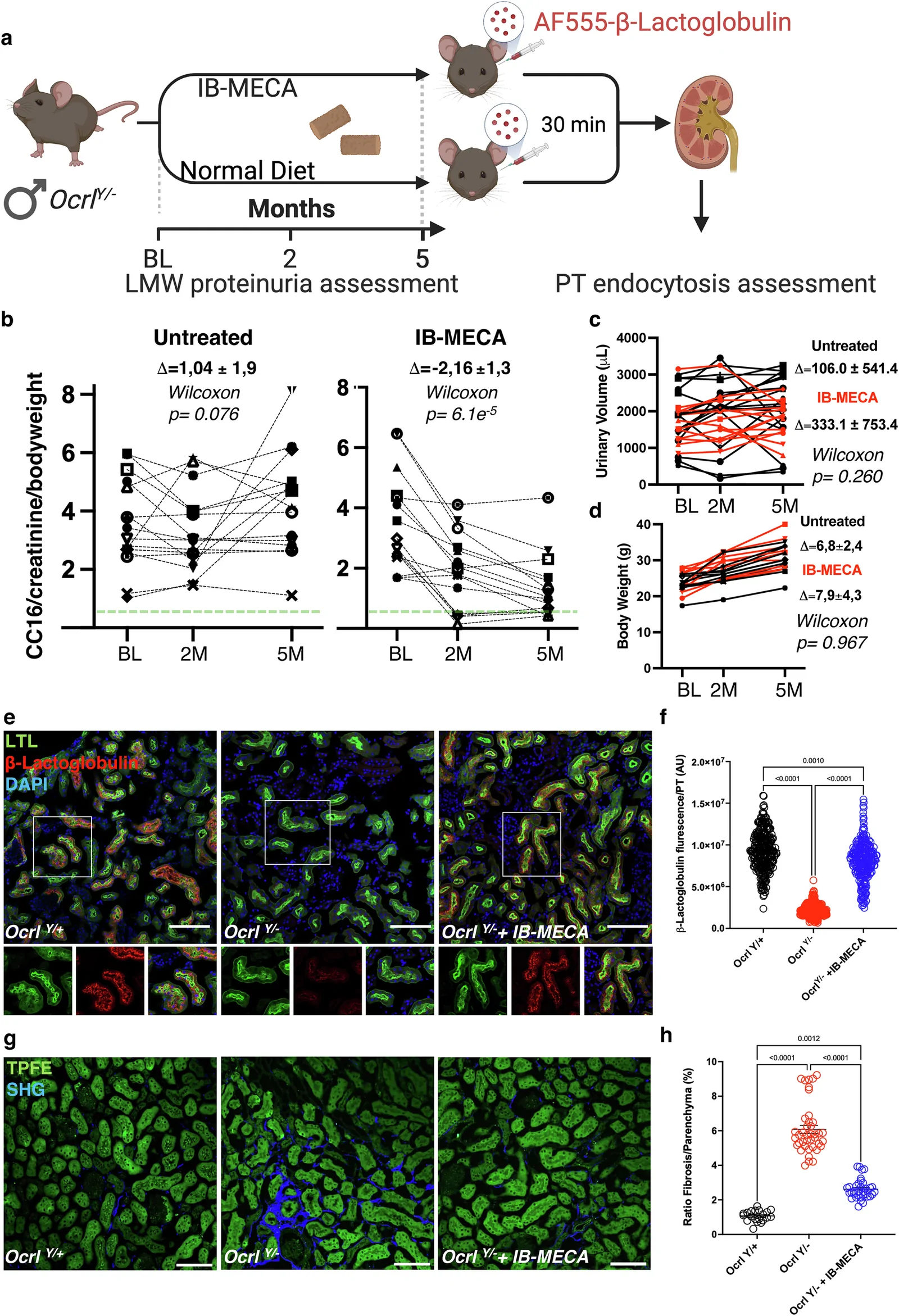
IB-MECA rescues LMW proteinuria and improves proximal-tubule protein reabsorption in *Ocrl*^*Y/−*^ mice. **a** Schematic of the study design. Male *Ocrl*^*Y/−*^ mice received a normal diet or chow containing IB-MECA for 5 months. Urine analysis for low molecular weight (LMW) proteinuria assessment was performed at baseline (BL), 2 months (2 M), and 5 months (5 M). After the last urine collection (5 M), the mice were intravenously injected (retroorbital injection) with Alexa Fluor 555-conjugated β-lactoglobulin (AF555-β-lactoglobulin). Thirty minutes after injection, the mice were sacrificed, and the kidneys were harvested for histological analysis. **b** LMW proteinuria was measured, and the amount of urinary Clara-Cell protein 16 (CC16) was normalized to creatinine and body weight (CC16/creatinine/body weight) in untreated and IB-MECA–treated *Ocrl*^*Y/−*^ mice at BL, 2 M, and 5 M. The green dashed line indicates the mean urinary CC16 level in age-matched WT (*Ocrl*^*Y/+*^*)* mice. **c** Urine volume was assessed in untreated and IB-MECA–treated *Ocrl*^*Y/−*^ mice at BL, 2 M, and 5 M. **d** Body weight of untreated and IB-MECA–treated *Ocrl*^*Y/−*^ mice at BL, 2 M, and 5 M. For **b**–**d**, each symbol represents an individual mouse. Statistical analysis was performed within groups by the Wilcoxon signed-rank test to compare BL vs 5 M. The exact *p* values for the untreated and IB-MECA-treated groups are reported in the figure, along with the Δ, which denotes the mean difference (5 M − BL) computed within the indicated cohort. Other timepoints (2 M) are shown descriptively. **e** Representative immunofluorescence analysis of kidney sections from *Ocrl*^Y/+^, *Ocrl*^*Y/−*^, and *Ocrl*^*Y/−*^+IB-MECA mice at 5 months after intravenous AF555–β-lactoglobulin injection. [Scale bars, 100 µm]. **f** Quantification of proximal-tubule LMW protein reabsorption (AF555–β-Lactoglobulin fluorescence) in *Ocrl*^*Y/+*^, *Ocrl*^*Y/−*^, and *Ocrl*^*Y/−*^+IB-MECA mice at 5 months. For each mouse, 50 proximal tubules were analyzed per mouse (*Ocrl*^*Y/+*^: *N* = 5; *Ocrl*^*Y/−*^: *N* = 15; *Ocrl*^*Y/−*^+IB-MECA: *N* = 15). Points represent values from individual tubules. Statistical analysis was performed by the Kruskal–Wallis test across the three independent groups with appropriate post hoc pairwise tests. **g** Assessment of tubulointerstitial fibrosis by multiphoton microscopy. Representative images of kidney cortex from WT (*Ocrl*^*Y/+*^), untreated *Ocrl*^*Y/−*^, and IB-MECA-treated *Ocrl*^*Y/−*^ mice are shown. Renal cortical parenchyma is shown in green by two-photon excited fluorescence (TPEF) autofluorescence, while the fibrillar collagen/fibrotic signal is shown in blue by second-harmonic generation (SHG). **h** Quantitative analysis shows the fibrosis/parenchyma ratio (SHG/TPEF), which was calculated for each acquired image. Each point represents a single image acquired from 2 *Ocrl*^*Y/+*^ mice, 4 untreated *Ocrl*^*Y/−*^ mice, and 4 IB-MECA-treated *Ocrl*^*Y/−*^ mice. [Scale bar, 100 µm]. Statistical analysis was performed using the Kruskal–Wallis test

Two-month-old *Ocrl*^*Y/-*^ mice received either standard chow or chow containing IB-MECA at 100 μg/kg/day for five months (Fig. 4a). LMW proteinuria was quantified by ELISA measurement of urinary Club Cell protein 16 (CC16), a sensitive marker of proximal tubular resorption, at baseline (BL) and after two (2 M) and five months (5 M) of IB-MECA treatment. IB-MECA administration resulted in a progressive, significant reduction in urinary CC16 levels, with decreases ranging from 48 to 80% compared to untreated *Ocrl*^*Y/-*^ mice (Fig. 4b and Supplementary Table 2), bringing proteinuria levels closer, and in some mice identical, to those measured in WT (*Ocrl*^*Y/+*^*)* mice. No changes in urinary volume and in the measured urinary and plasma parameters (Fig. 4c and Supplementary Table 2) or mouse weight (Fig. 4d) were observed, consistent with IB-MECA’s safety profile, also established in prior studies.^36,37^

To determine whether the reduction in LMW proteinuria reflected an improvement in receptor-mediated endocytosis within PTCs, we analyzed the uptake of AlexaFluor (AF)-555–labeled β-lactoglobulin in vivo.^22,46^ Following intravenous injection, this fluorescent tracer is freely filtered by glomeruli and taken up by PTCs through endocytosis. The tracer was administered to *Ocrl*^*Y/+*^ and *Ocrl*^*Y/-*^ mice, either untreated or treated with IB-MECA (Fig. 4a). Thirty minutes post-injection, a strong fluorescence signal was evident throughout most of the proximal tubules in *Ocrl*^*Y/+*^ mice but was markedly diminished in untreated *Ocrl*^*Y/-*^ kidneys, which is consistent with previously reported findings.^22^ Strikingly, IB-MECA–treated *Ocrl*^*Y/-*^ mice displayed robust proximal tubule uptake comparable to that observed in *Ocrl*^*Y/+*^ mice (Fig. 4e, f).

As mentioned above, tubulointerstitial fibrosis (TIF) is a key determinant of CKD progression in LS patients.^13^ We therefore evaluated whether IB-MECA could counteract the development of TIF in *Ocrl*^*Y/-*^ mice. TIF was quantified by multiphoton microscopy, which enables label-free detection of type I collagen fibers in the renal cortical parenchyma and thereby provides a direct readout of interstitial matrix remodeling. In comparison with *Ocrl*^*Y/+*^ mice, *Ocrl*^*Y/-*^ mice displayed widespread peritubular collagen accumulation within the cortical interstitium in the absence of gross alterations in global kidney architecture (Fig. 4g, h). Notably, IB-MECA treatment, while not affecting overall parenchymal organization, markedly reduced type I collagen deposition in *Ocrl*^*Y/-*^ mice (Fig. 4g, h). Interestingly, these in vivo findings mirror our observation that IB-MECA reduces extracellular matrix components in kidney organoids (Supplementary Fig. 4b) and collectively support an anti-fibrotic role of A3 adenosine receptor activation, which is consistent with previous reports.^47,48^

The data obtained in *Ocrl*^*Y/-*^ mice, while confirming the excellent safety profile of IB-MECA, underscore its ability not only to reduce proteinuria but also to potentially attenuate kidney disease progression.

Taken together, these results demonstrate that IB-MECA effectively restores the endocytic and reabsorptive functions impaired by OCRL deficiency across multiple LS models—including cultured cells, kidney organoids, and mice—highlighting its strong translational potential. With IB-MECA now patented for LS therapy (WO2025011999) and progressing to a phase II clinical trial in LS patients (NCT07410455), determining the molecular mechanism underlying its ability to rescue PTC reabsorptive capacity has become a critical next step in validating its therapeutic efficacy.

### Mechanism of action of IB-MECA in correcting endocytic defects in LS

IB-MECA is a selective agonist of A3AR, a G protein-coupled receptor (GPCR) encoded by the ADORA3 gene and expressed in several tissues, including kidney proximal tubules.^44,49–53^

**Fig. 5:**
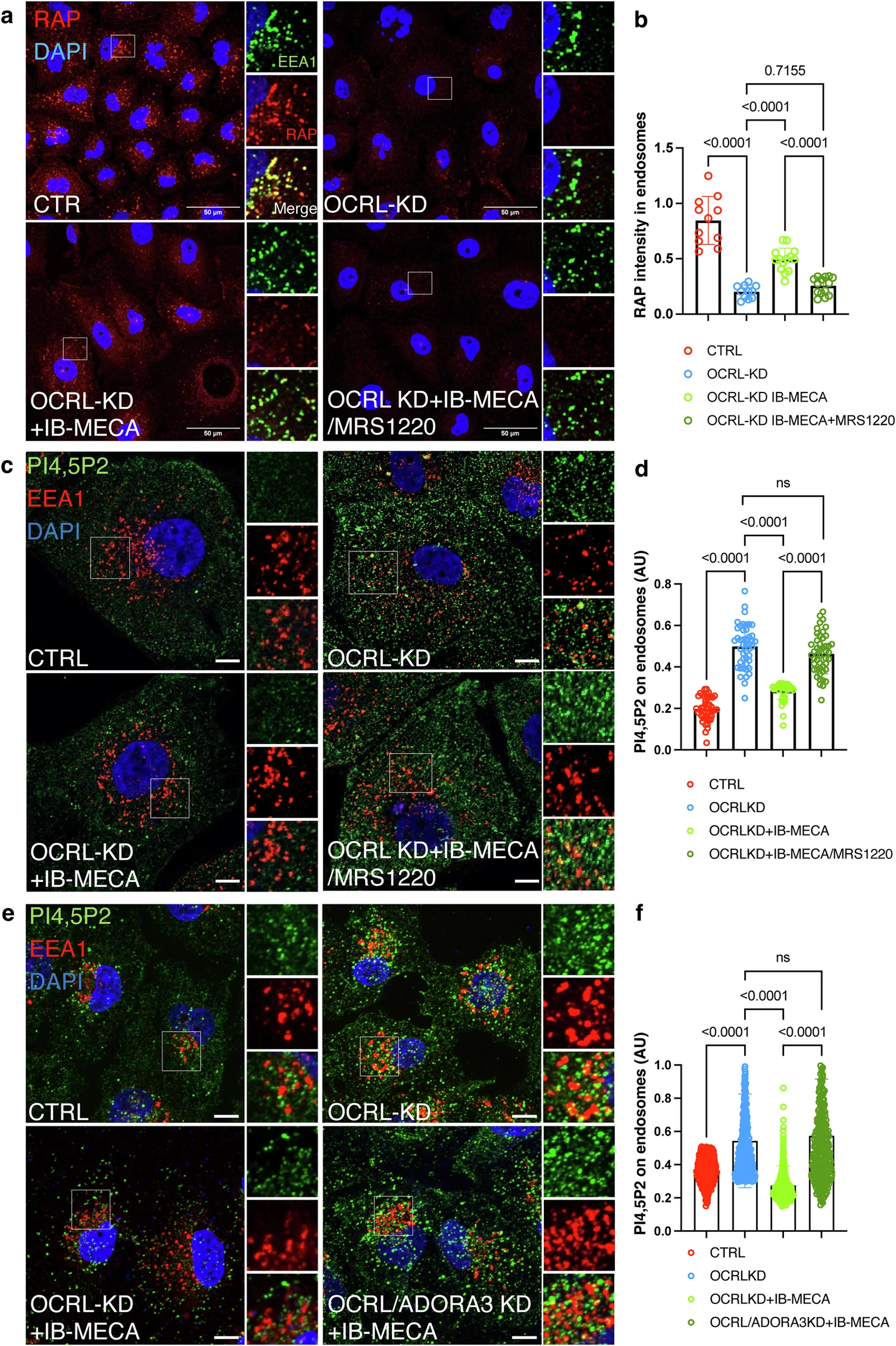
IB-MECA decreases endosomal PI(4,5)P_2_ levels in OCRL-depleted cells via A3AR. **a** Representative images of RAP (red) uptake in control (CTRL) and OCRL-knockdown (OCRL-KD) HK-2 cells either untreated or treated for 16 h with IB-MECA (250 nM) or with IB-MECA + the A3AR antagonist (MRS1220, 16 h, 250 nM). Anti-EEA1 (green) was used to label early endosomes and is shown only in the magnified boxed areas. The nuclei were labeled with DAPI. [Scale bars, 50 µm.] **b** Quantification of RAP uptake under the conditions in (**a**). Points represent fields with at least 20 cells from 3 independent biological replicates (*n* > 200, *N* = 3). Statistical analysis was performed by the Kruskal–Wallis test with Dunn’s multiple comparison post hoc test, with exact *p* values shown on the plot. **c** Representative images of CTRL and OCRL-KD HK-2 cells either left untreated or treated for 16 h with IB-MECA (250 nM) or with IB-MECA + the A3AR antagonist (MRS1220, 16 h, 250 nM) labeled with PI(4,5)P_2_ (green) and EEA1 (red). [Scale bars, 10 µm]. **d** Quantification of endosomal PI(4,5)P_2_ intensity (colocalizing with EEA1) under the conditions in (**c**). Points are per-cell with at least 100 cells from 3 independent biological replicates (*n* > 300, *N* = 3). Statistical analysis was performed by the Kruskal–Wallis test with Dunn’s multiple comparison post hoc test, with exact *p* values shown on the plot. **e** Representative images of CTRL, OCRL-KD, and OCRL/ADORA3 double-KD HK-2 cells either untreated or treated for 16 h with IB-MECA (250 nM) and labeled with PI(4,5)P_2_ (green) and EEA1 (red). [Scale bars, 10 µm]. **f** Quantification of endosomal PI(4,5)P_2_ intensity (colocalizing with EEA1) under the conditions in (**e**). Points are per-cell with at least 100 cells from 3 independent biological replicates (*n* > 300, *N* = 3). Statistical analysis was performed by the Kruskal–Wallis test with Dunn’s multiple comparison post hoc test, with exact *p* values shown on the plot

To determine whether the rescue of endocytic defects caused by the loss of OCRL requires A3AR activation, the selective A3AR antagonist MRS1220 was used.^54^ MRS1220 abolished the ability of IB‑MECA to restore receptor‑mediated endocytosis in OCRL-KD HK-2 cells (Fig. 5a, b), indicating that IB‑MECA acts through A3AR. Consistent with this, and as reported above, we found that ADORA3/A3AR is expressed in all kidney models that respond to IB‑MECA, including HK-2 cells, human kidney organoids, and mouse proximal tubule cells (Supplementary Table 3, Fig. 3f–h, Supplementary Fig. 3, Supplementary Data 4, 5). Moreover, ADORA3 mRNA and A3AR protein are expressed in the human kidney (Supplementary Table 3).^44,49–53^

A3AR signals via both Gαi and Gαq proteins, with Gαi inhibiting adenylyl cyclase and Gαq, together with Gβγ, stimulating phospholipase C β (PLCβ).^55–59^ PLCβ selectively hydrolyzes PI(4,5)P_2_ to generate diacylglycerol (DAG) and inositol triphosphate (IP3),^60^ providing an alternative route for PI(4,5)P_2_ consumption distinct from OCRL‑mediated dephosphorylation.

Guided by the notion that endocytic defects in OCRL‑depleted cells are driven mainly by aberrant ectopic accumulation of PI(4,5)P_2_ on early endosomes, we next investigated the mechanism of IB‑MECA–mediated rescue by examining its effects on the levels of PI(4,5)P_2_, the OCRL substrate. As expected, OCRL‑deficient cells displayed marked accumulation of PI(4,5)P_2_ in early endosomes (Fig. 5c, d). Strikingly, IB‑MECA treatment reduced endosomal PI(4,5)P_2_ in OCRL‑KD cells to near‑control levels. This PI(4,5)P2-lowering effect, which was paralleled by a reduction in endosomal actin (Supplementary Fig. 5a, b), was mediated by A3AR, as it was blunted by ADORA3 knockdown and MRS1220 (Fig. 5c–f; Supplementary Fig. 5a–c).

**Fig. 6:**
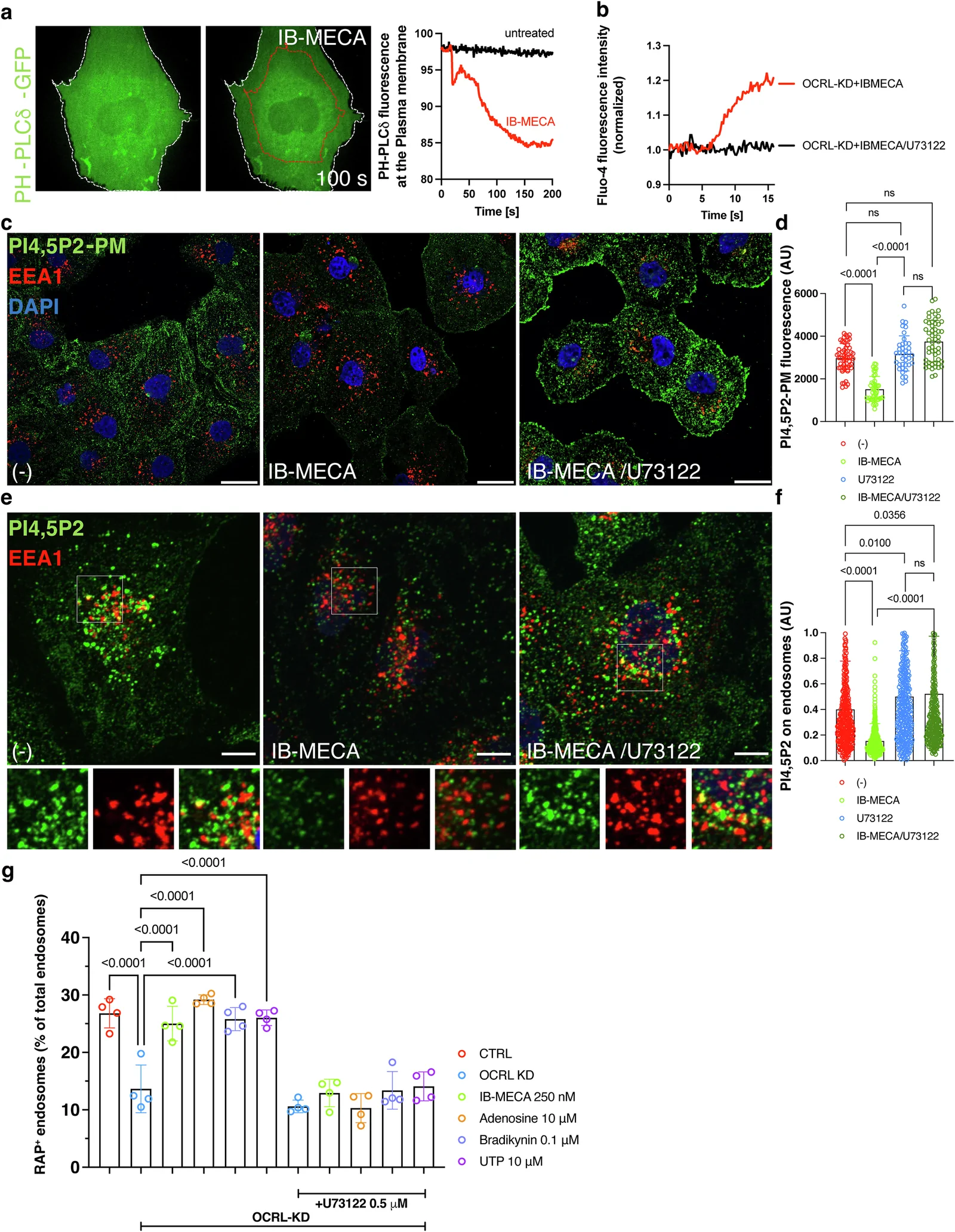
IB-MECA and other PLC-coupled GPCR agonists counteract endosomal PI(4,5)P_2_ accumulation and restore endocytic function in OCRL-depleted cells through PLC signaling. Acute stimulation of A3AR with IB-MECA induces PLC activation at the plasma membrane, as indicated by **a** the release of the PI(4,5)P_2_ probe (PH-PLCδ) from the plasma membrane to the cytosol in OCRL-KD HK-2 cells and by **b** the transient increase in cytosolic Ca^2+^ (prevented by coadministration of U73122, 10 µM) in OCRL-KD HK-2 cells loaded with the Ca^2+^-sensitive dye Fluo-4. **c** Representative images of OCRL-KD HK-2 cells that were either left untreated (-) or treated for 16 h with IB-MECA (250 nM) or with IB-MECA + the PLC inhibitor (U73122, 16 h, 0.5 µM), labeled for the plasma membrane pool of PI(4,5)P_2_ (green) and EEA1 (red). [Scale bars, 20 µm]. **d** Quantification of plasma membrane PI(4,5)P_2_ intensity for the conditions in (**c**). Points are per-cell with at least 100 cells from 3 independent biological replicates (*n* > 300, *N* = 3). Statistical analysis was performed by the Kruskal–Wallis test with Dunn’s multiple comparison post hoc test, with exact *P* values shown on the plot. **e** Representative images of OCRL-KD HK-2 cells that were either left untreated or treated for 16 h with IB-MECA (250 nM) or with IB-MECA + the PLC inhibitor (U73122, 16 h, 0.5 µM) labeled with endosomal PI(4,5)P_2_ (green) and EEA1 (red). [Scale bars, 10 µm]. **f** Quantification of endosomal PI(4,5)P_2_ intensity (colocalizing with EEA1) under the conditions in (**e**). A bar plot was generated with per-endosome/per-cell values for at least 100 cells from 3 independent biological replicates (*n* > 300, *N* = 3). Statistical analysis was performed by the Kruskal–Wallis test with Dunn’s multiple comparison post hoc test, with exact *p* values shown on the plot. **g** Activation of PLC-coupled GPCRs restores endocytic activity in OCRL-KD cells, and this rescue is blocked by U73122. Quantitative analysis of RAP uptake in OCRL-KD HK-2 cells. RAP uptake was measured as the number of EEA1-positive endosomes per cell that were also positive for RAP-GST in control (CTRL) and in OCRL-KD HK-2 cells either untreated (-) or stimulated with IB-MECA, adenosine, bradykinin, or UTP, either alone or in combination with the PLC inhibitor U73122. Data were obtained by high-content imaging (*n* ≥ 450 cells per condition; *N* = 4 independent biological replicates, with 4 fields per condition and at least 50 cells per field) and are presented as the mean ± SEM. Statistical significance was assessed by one-way ANOVA followed by Tukey’s post hoc test

Given the known coupling of A3AR to PLC, we hypothesized that the reduction in endosomal PI(4,5)P_2_ by IB‑MECA involves PLC activation at the plasma membrane (PM). To test this hypothesis, we first assessed the impact of IB‑MECA on PM PI(4,5)P_2_ and on cytosolic Ca^2+^ (as a readout of IP3 production) in both OCRL‑KD (Fig. 6a, b) and control HK-2 cells (Supplementary Fig. 6a, b). Fluorescent labeling with a PH‑PLCδ probe, which selectively binds PI(4,5)P_2_, revealed that IB‑MECA induced dissociation of the probe from the PM, which is consistent with PLC activation and PI(4,5)P_2_ hydrolysis (Fig. 6a). Concomitantly, in both OCRL‑KD cells (Fig. 6b) and control cells (Supplementary Fig. 6b) loaded with the Ca^2+^‑sensitive dye Fluo‑4, IB‑MECA triggered a transient cytosolic Ca^2+^ spike that was sensitive to the PLC inhibitor U73122,^61^ confirming PLC activation.

We then asked whether the IB‑MECA–induced decrease in endosomal PI(4,5)P_2_ depends on PLC activity at the PM. PI(4,5)P_2_ levels at the PM were quantified in control (Supplementary Fig. 6c, d) and OCRL‑KD cells (Fig. 6c, d). Consistent with the findings of previous reports^6^, the PM PI(4,5)P_2_ levels were higher in the OCRL‑KD cells than in the control cells (Supplementary Fig. 6c, d), yet they were efficiently reduced by IB‑MECA treatment (Fig. 6c, d). This effect was abolished by U73122, demonstrating that the decrease in PM PI(4,5)P_2_ is PLC dependent (Fig. 6c, d). Lowering the level of PM PI(4,5)P_2_ also led to a reduction in endosomal PI(4,5)P_2_, and this effect was likewise prevented by U73122 (Fig. 6e, f; Supplementary Fig. 6e, f). These observations indicate that, particularly in the absence of OCRL, the endosomal PI(4,5)P_2_ pool depends not only on local synthesis but also, to a large extent, on PI(4,5)P_2_ supplied from the PM.

Finally, we examined whether this mechanism extends to other PLC‑coupled GPCRs. Stimulation of purinergic (P2Y) receptors elicited PLC‑dependent Ca^2+^ release^62^ in OCRL‑KD and control cells (Supplementary Fig. 7a), and stimulation of bradykinin and purinergic receptors, as well as adenosine receptors other than A3, restored endocytic activity in OCRL‑KD cells (Fig. 6g). With respect to all the ligands, the rescue effect was PLC dependent, as cotreatment with U73122 blocked this effect (Fig. 6g; Supplementary Fig. 7a, b). UTP stimulation phenocopied IB‑MECA, reducing endosomal PI(4,5)P_2_ to a similar extent (Supplementary Fig. 7c, d). Together, these findings show that activation of PLC‑coupled cell‑surface GPCRs modulates endosomal PI(4,5)P_2_ homeostasis in OCRL‑deficient cells, highlight OCRL as the principal 5‑phosphatase orchestrating the endocytic PI switch, and reveal plasma‑membrane receptor signaling as a potential strategy to correct endosomal phosphoinositide imbalances in this context.

## Discussion

A detailed understanding of phosphoinositide regulation and endosomal trafficking in LS enabled us to use basic cell biology to design a screening strategy that ultimately identified a targeted therapeutic agent.

Pathological LS features—including impaired recycling of megalin and M6PR, abnormal actin organization, and defective autophagy—are causally tied to OCRL-dependent control of phosphoinositides.^6,16,22^ Previous work, including our own, established that OCRL loss drives PI(4,5)P_2_ accumulation in endosomal membranes, resulting in disrupted receptor recycling, perturbed actin dynamics, and impaired autophagy.^16–18,63–72^ To directly address these molecular defects, our phenotypic screening pipeline spanned engineered OCRL-KD cell lines, patient-derived kidney cells, kidney organoids, and *Ocrl*^*Y/-*^ mice, always prioritizing disease-relevant functional readouts. Among the 1,280 tested compounds, IB-MECA was identified as a highly effective corrector. Mechanistically, we showed that IB-MECA, which acts through A3AR, induces PLC-dependent PI(4,5)P_2_ consumption. IB-MECA treatment normalized endosomal PI(4,5)P_2_, restored receptor recycling and protein reabsorption, and corrected autophagic impairment across all the models. Importantly, and broadening the relevance of our findings, the imbalance in endosomal PI(4,5)P_2_ and in endocytic receptor trafficking was reproduced with other PLC-coupled GPCR agonists. A key conceptual advance from these findings is the demonstration that PI(4,5)P_2_ overload on endosomes in LS is sustained by plasma membrane PI(4,5)P_2_ pools, revealing a disease-relevant plasma membrane–endosome phosphoinositide crosstalk. This principle appears to be conserved across species, as Drosophila and zebrafish studies have shown that PLC chemical activators can counteract endosomal PI(4,5)P_2_ overload and trafficking defects caused by the disruption of OCRL homologs.^73^

The therapeutic relevance of IB-MECA (piclidenoson) is strengthened by its advanced clinical development. Large-scale trials, including phase III studies, in patients with moderate-to-severe plaque psoriasis have demonstrated that piclidenoson achieves clinically meaningful responses with excellent safety and tolerability profiles. Specifically, the CONFORT-1 study (ClinicalTrials.gov identifier: NCT03168256) included 529 adult patients with moderate-to-severe chronic plaque psoriasis.^37^ The primary endpoint (i.e., a > 75% reduction in the Psoriasis Area and Severity Index [PASI] score by week 16) was achieved at a dosage of 3 mg twice daily, accompanied by excellent safety and tolerability profiles, as evidenced by comparable rates of adverse events between the treatment and placebo groups.^37^ Phase II and IIb studies in rheumatoid arthritis, while not superior to methotrexate for efficacy, have consistently confirmed a very favorable safety profile, including low rates of treatment-emergent adverse events (ClinicalTrials.gov identifiers: NCT00556894 and NCT01034306).^74,75^ In our LS models, robust efficacy of IB-MECA was observed in cell systems, organoids, and *Ocrl*^*Y/−*^ mice, in which long-term treatment led to a sustained reduction in proteinuria without detectable adverse effects or loss of therapeutic response, aligning the preclinical benefit with the reassuring human safety record. These converging lines of evidence provide a strong rationale for evaluating piclidenoson in LS patients. A phase II clinical study has therefore been designed (NCT07410455) and is currently under review by health authorities, with improvement in low–molecular weight proteinuria after six months of treatment as the primary endpoint, which is used as a proxy for endolysosomal function in renal proximal tubule cells.

Conceptually, this work forges a direct link between cell biological dissection of phosphoinositide control of membrane trafficking and therapeutic intervention. The finding that receptor-driven, PLC-mediated PI(4,5)P_2_ hydrolysis at the plasma membrane can “drain” pathologically expanded endosomal PI(4,5)P_2_ pools and restore multiple downstream processes defines a novel therapeutic paradigm for inborn errors of phosphoinositide metabolism on the basis of an actionable ligand–receptor approach. In this context, A3AR, which is expressed at relatively low levels in the kidney compared with other GPCRs (Supplementary Data 5), offers a way to modestly engage PLC-dependent PI(4,5)P₂ turnover sufficient to correct OCRL-linked defects while avoiding the broad second-messenger surges triggered by more abundant GPCRs. This strategy allows fine-tuning of phosphoinositide levels while preserving the intrinsic activities of PI kinases. In parallel, recent studies^46^ have used a phosphoinositide 3-kinase (PI3K) inhibitor and anti-cancer agent alpelisib in an *Ocrl*^*Y/−*^ mouse model to correct phosphoinositide and actin imbalances, further supporting the idea that targeting phosphoinositide pathways can restore actin organization and endocytic function. Together, these lines of evidence strengthen the link between altered phosphoinositide metabolism, actin cytoskeletal disturbances, and endocytosis in LS and point to a broader therapeutic framework in which modulation of phosphoinositide signaling—through receptor-based approaches or kinase-targeting strategies—offers promising routes for disease correction.

Although this study focuses on renal manifestations, LS is a multisystem disorder with prominent central nervous system (CNS) involvement. Emerging evidence now suggests that restoring PI(4,5)P_2_ levels via A3AR activation in LS may also be beneficial in the brain. Studies in patient-derived brain organoids have shown that lowering the level of PI(4,5)P_2_ rescues the neurodevelopmental defects caused by OCRL loss, directly implicating PI(4,5)P_2_ dysregulation in the LS CNS.^76^ In parallel, IB-MECA has very recently been shown to exert neuroprotective effects through A3AR stimulation in preclinical models.^77^ Together, these observations provide a compelling rationale to extend a mechanism-based, phosphoinositide-targeted strategy developed in kidney models to neurological manifestations of LS and, more broadly, to other phosphoinositide-driven disorders of intracellular trafficking. From this perspective, the combination of high-content imaging, organoid modeling, multi-parametric screening, and rigorous cross-species validation does not simply support our current findings; it defines a generalizable platform for turning such mechanistic insights into targeted therapies. Ongoing work will leverage this platform to elucidate tissue-specific mechanisms and drive clinical translation, with the overarching goal of delivering effective, mechanism-based treatments for LS and related disorders of intracellular trafficking.

## Materials and methods

### Ethics declarations

All animal experiments were approved by the Italian Ministry of Health, Department for Veterinary Public Health, Nutrition and Food Safety Directorate-General for Animal Health and Veterinary Medicinal Products, Office VI – Animal Welfare (permission number CE571.14). HeLa and HK-2 cells were obtained from the ATCC and confirmed to be mycoplasma free. Primary proximal tubule epithelial cells (PTCs) were kindly provided by Elena Levtchenko (Amsterdam UMC) and were tested for mycoplasma contamination. Wild-type (WT) and OCRL-knockout (OCRL-KO) induced pluripotent stem cell (iPSC) lines were obtained from Herbert Lachman (Albert Einstein College of Medicine). The study involving the original human donors, including the relevant consent procedures, was approved by the Albert Einstein College of Medicine Institutional Review Board (approval no. 2023-15561). All the cell lines were confirmed to be mycoplasma free and were handled in accordance with institutional biosafety regulations and standard cell culture practices.

### Antibodies and reagents

All antibodies, reagents, dyes, media, kits, and consumables used in this study were obtained from commercial sources or through collaborations as specified below, while the reagents used only in specific experimental procedures are detailed in the corresponding Methods section. The primary antibodies used were M6PR (Novus, NB-300-514), EEA1 (mouse; BD Biosciences, 610456), EEA1 (rabbit; Cell Signaling Technology, C45B10), NPHS1/nephrin (Progen, GP-N2), LRP2/megalin (Abcam, ab76969), anti-adenosine A3 receptor antibody (AlomoneLabs, AAR-004), Na⁺/K⁺-ATPase (Abcam, ab7671), p62/SQSTM1 (Progen, GP62-C), LC3 (Novus, NB100-2220), and PI(4,5)P2/PIP2 (Echelon Biosciences, Z-P045). The following secondary antibodies (all from Invitrogen) were used: goat anti-mouse IgM (μ chain), Alexa Fluor 488 (A21042), donkey anti-mouse IgG (H + L), Alexa Fluor 568 (A10037), donkey anti-mouse IgG (H + L), Alexa Fluor 647 (A31571), donkey anti-rabbit IgG (H + L), Alexa Fluor 488 (A21206), donkey anti-rabbit IgG (H + L), Alexa Fluor 568 (A10042), and goat anti–guinea pig IgG (H + L) Alexa Fluor 647 (A21450). The following fluorescent tracers/dye were used: BSA–Alexa Fluor 555 (Invitrogen, A34786), Dextran–Alexa Fluor 647 (Invitrogen, D22914), Alexa Fluor Phalloidin (Invitrogen, A22287), Hoechst 33342 (Invitrogen, H3570), Fluo-4/AM (Invitrogen, F14217), and LTL-FITC (Invitrogen, L32480). Small molecules and differentiation reagents: ROCK inhibitor Y-27632 (STEMCELL Technologies, 72304), CHIR99021 (STEMCELL Technologies, 72054), tetracycline (Merck, T7660), hydrocortisone (Merck, H0888), human EGF (Merck, E1235), tri-iodothyronine (Merck, T5513), β-mercaptoethanol (Thermo Fisher, 21985023), and KnockOut™ Serum Replacement (Gibco, 10828028), IB-MECA (Tocris, 1066), UTP (Merck, U7865), bradykinin (Merck, 05-0500), adenosine (Merck, A9251), MRS1220 (Tocris, 1217) and U73122 (Tocris, 1268). The culture media, matrices, buffers, and enzymes used were DMEM/F-12 (Gibco, 21331-046), low-glucose DMEM supplemented with pyruvate (Gibco, 11885084), L-glutamine (Gibco, 25030-081), penicillin/streptomycin (Gibco, 15140-122), MEM supplemented with nonessential amino acids (Gibco, 11140050), GlutaMAX (Gibco, 35050061), HEPES (Gibco, 15630080), Plasmocin (InvivoGen, ant-mpt), fetal calf serum (Euroclone, ECS0186L), ITS (Insulin-Transferrin-Selenium, Sigma‒Aldrich, I1884-1VL), insulin-transferrin-selenium-ethanolamine (ITS-E; Gibco, 51500056), mTeSR1 Basal medium (STEMCELL Technologies, 85850), mTeSR1 Plus Basal medium (STEMCELL Technologies, 100-0276), TeSR-E5 medium (STEMCELL Technologies, 05916), Geltrex™ (cat. A1413301), DPBS 1× (Euroclone, ECB4004), and PBS supplemented with Ca²⁺/Mg²⁺ (Euroclone, ECB4053L), EDTA (Invitrogen, 15575-038), Dispase (STEMCELL Technologies, 07923), Accutase (Millipore, SCR005), Anti-Adherence Rinsing Solution (STEMCELL Technologies, 07010), polyvinyl alcohol (PVA; Merck, 141355), chemically defined lipid concentrate (Gibco, 11905031), digitonin (Calbiochem, 3004105), and goat serum (Gibco, 16210-072). Transfection/selection and molecular biology kits included BLOCK-iT™ Inducible H1 RNAi Entry Vector Kit (Life Technologies, K4925-00), Lipofectamine 2000 (Life Technologies, 11668500), Zeocin (Gibco, R25001), blasticidin (Gibco, A1113903), Lipofectamine™ RNAiMAX (Thermo Fisher Scientific, 13778150), an RNA extraction kit (Qiagen, 74134), and SuperScript™ IV VILO™ Master Mix (Invitrogen, 11756050). Fixation/mounting and imaging consumables: paraformaldehyde (Electron Microscopy Sciences, 157-8-100), Mowiol (Calbiochem, 475904), glass microscope slides (Epredia, 16247920), and imaging spacers (Invitrogen, S24737). Additional assays and in vivo reagents included an Alexa Fluor™ 555 Protein Labeling Kit (Life Technologies, A20174), an ELISA kit for CC16 (BIOMATIK, EKU03200), a control diet (#4RF21, Mucedola), and IB-MECA-containing chow (#4RF21 supplemented with 0.75 mg/kg IB-MECA, Mucedola).

### Cell lines

HeLa cells stably transfected with an expression cassette containing a shRNA-OCRL under a tetracycline (Tet)-regulated promoter were generated using the BLOCK-iT Inducible H1 RNAi Entry Vector Kit (Life Technologies, K4925-00). HeLa T-REx cells were transfected with shRNA-OCRL (TAGCCACTTCAGATGTGTTCAAGAAGCAG) pENTR/JH1/TO vectors using Lipofectamine 2000 (Life Technologies, 11668500) and selected with 300 µg/mL zeocin (Gibco, R25001) and 5 µg/mL blasticidin (Gibco, A1113903). After three weeks of selection, eight colonies were obtained and screened for OCRL knockdown after tetracycline (1 mg/mL) treatment for 72 h and 96 h. A single HeLa shRNA-OCRL clone showing an 80% reduction in OCRL expression was selected for further analysis (Supplementary Fig. 1a). HeLa cells were grown in DMEM supplemented with 2 mM L-glutamine, 50 IU/mL penicillin, 50 mg/mL streptomycin, and 10% fetal calf serum.

HK-2 (ATCC CRL-2190) cells were grown in DMEM/F-12 (Gibco, 21331-046) supplemented with 1% ITS, 2 mM L-glutamine, 50 IU/mL penicillin, 50 mg/mL streptomycin, and 5% fetal calf serum.

PTCs from healthy donors and from Lowe patients^16^ were grown in DMEM/F-12 supplemented with 10% fetal calf serum, 50 IU/mL penicillin, 50 mg/mL streptomycin, 1% ITS, 36 ng/mL hydrocortisone, 10 ng/mL human EGF, and 40 pg/mL triiodothyronine.

Human induced pluripotent stem cell (iPSC) lines generated from human peripheral blood CD34+ cells were previously characterized.^38,39^ iPSCs were cultured at 37 °C and 5% CO₂ and maintained in mTeSR1 basal medium supplemented with 1× supplement or in mTeSR1 plus basal medium. The culture plates were coated with 100× Geltrex, thawed on ice and diluted to a final concentration of 1× in cold (4 °C) DMEM/F-12. Cells were passaged at 60–70% confluence by removing the spent medium, after which they were washed twice with 5 mL of DPBS 1× and treated with 3 mL of 0.5 µM EDTA in 1× DPBS for 3–5 min at room temperature as a gentle dissociation agent. EDTA was then removed, and adherent cells were collected in iPSC maintenance medium supplemented with 1 µM ROCKi (Y-27632), gently broken into small colonies using a 5 mL stripette, and reseeded at a 1:5 to 1:10 ratio onto fresh Geltrex-coated plates. The ROCKi was removed after 24 h, and the cells were cultured until they reached ~60% confluence (48–72 h post passage) prior to initiating differentiation.

### siRNA transfection

HK-2 cells were plated in 6-well plates (150,000 cells/well) or in 24-well plates (15,000 cells/well) and treated with 30 nM OCRL siRNA (5’-GAUUACUUCUUGACUAUCA-3’, 5’-GAAAGGAUCAGUGUCGAUA-3’, 5’-CUCCCGCAGUUGAACAUCA-3’) or 35 nM ADORA3 siRNA (5’-GAACUUAUCUAACUCCAAA-3’, 5’-CGUCUAUGCCUAUAAAAUA-3’, 5’-CAAACUCAGUCUGAACUUA-3’) for 96 h using Lipofectamine™ RNAiMAX.

### RNA extraction, reverse transcription, and quantitative real-time PCR

ADORA3 expression in HK-2 cells transfected with siRNA and in WT and OCRL-KO kidney organoids was measured using RT‒qPCR. RNA was extracted using an RNA extraction kit (Qiagen, 74134), and reverse transcription was performed with SuperScript™ IV VILO™ Master Mix (Invitrogen, 11756050) following the manufacturer’s instructions. DNA amplification was carried out with TaqMan™ Assay Probes specific for ADORA3 (Hs04194761_s1) and GAPDH (Hs03929097_g1) using TaqMan® Fast Advanced Master Mix (Applied Biosystems) on a LightCycler 480-II Real-Time PCR System (Roche).

### High-content analysis of the distribution of mannose 6-phosphate receptor

Automated analysis of M6PR distribution in OCRL-KD HeLa cells was performed as previously described.^16^ HeLa T-REx shRNA-OCRL cells were seeded in μClear black 96-well plates (Greiner Bio-One), left untreated (CTRL) or treated with 1 µg/mL tetracycline for 72 h (OCRL-KD), fixed in 4% paraformaldehyde for 8 min, washed in PBS, and stained with anti-M6PR antibody and Hoechst 33342. Images were acquired on a Scan^R automated epifluorescence microscope (Olympus) using a 20× UPlanApo objective (NA 0.7) and analyzed with Scan^R software in cell-based mode. Nuclei were segmented using fixed intensity and size thresholds, excluding irregular nuclei. In the M6PR channel, sub-objects were defined by an intensity threshold of 800 and a size range of 4–50 pixels, and their number and localization were quantified. Perinuclear and peripheral regions were generated from nuclear masks by dilation (0–40 pixels, perinuclear; 40–100 pixels, peripheral). Four subpopulations were defined from perinuclear and peripheral sub-object counts: (1) perinuclear continuous structures; (2) peripheral spots; (3) perinuclear fragmentation plus peripheral spots; and (4) perinuclear fragmentation. To assess the assay performance, a Z’ score was calculated to determine the separation between the untreated and tetracycline-treated controls. Z’ is defined as Z’ = 1 − (3(σ₊ + σ₋))/|μ₊ − μ₋|where μ and σ represent the mean and standard deviation of positive (+) and negative (–) controls, respectively. With respect to the M6PR assay, the Z’ score was 0.71, indicating that the M6PR assay is suitable for high-content screening.^78^

### High-content library screening: primary assay

High-content screening (HCS) was performed using the HeLa shRNA-OCRL clone seeded in μClear black microplates (PerkinElmer) in the absence (CTRL) or presence of tetracycline (1 µg/mL; OCRL-KD). After 72 h, the cells were treated for 16 h with compounds from the LOPAC library (Sigma‒Aldrich) at 10 µM. The cells were then fixed for 10 min in 4% PFA in PBS, permeabilized in blocking buffer (0.05% saponin, 0.5% BSA, 50 mM NH₄Cl in PBS), incubated with anti-CI-M6PR^16^ followed by Alexa Fluor-conjugated secondary antibodies and DAPI, and imaged and analyzed with the Scan^R platform as described above. The percentage of cells displaying perinuclear continuous M6PR was used as the primary readout. For each plate, the compound responses were first normalized using a linear rescue-scale transformation: normalized value = (X − μKD)/(μCTRL − μKD), where X is the value measured in the compound-treated well, μCTRL is the mean of the untreated control wells, and μKD is the mean of the tetracycline-treated OCRL-KD wells. This transformation assigns a value of 0 to OCRL-KD wells and 1 to CTRL wells, whereas compound-treated wells can assume values between 0 and 1 (partial rescue), >1 (overrescue), or <0 (further impairment). To rank compounds according to their deviation from the negative control population, z scores were calculated in parallel from the raw readout values using the OCRL-KD mean and standard deviation according to the formula z = (x − μKD)/σKD, where x is the raw value measured for each compound-treated well, μKD is the mean of tetracycline-treated OCRL-KD wells, and σKD is the corresponding standard deviation. With this approach, the positive control (CTRL) corresponded to a z score of 3.17. Compounds were then ordered from highest to lowest z scores and plotted to visualize the distribution of activity across the screened library.

### RAP–GST uptake assay

HK-2 and PTC cells at ~60% confluence were serum-starved for 1 h at 37 °C in DMEM/F-12 lacking FBS. The cells were incubated for 30 min at 4 °C in DMEM/F-12 (without FBS) supplemented with 0.5 µg/mL RAP–GST to allow ligand binding. Excess RAP–GST was removed by changing the medium, and the cells were shifted to 37 °C for 5 min to allow receptor-mediated endocytosis. The noninternalized ligand was removed by acid washing (150 mM NaCl and 10 mM acetic acid in water) for 45 s at room temperature, followed by three washes with PBS containing Ca²⁺/Mg²⁺. Cells were fixed in 4% PFA for 7 min at room temperature and processed for immunofluorescence as described below.

### High-content RAP–GST internalization assay

CTRL and OCRL-KD HK-2 cells (6000 cells/well) were seeded in μClear black 96-well plates (PerkinElmer). Cells were treated for 16 h at 37 °C with selected LOPAC compounds at 1, 3, or 10 µM, serum-starved for 1 h, and then incubated for 1 h at 4 °C with 2.5 µg/mL GST–RAP, followed by 5 min at 37 °C to allow internalization. After being washed with ice-cold complete medium, surface-bound GST–RAP was removed by a 45 s incubation with acid wash solution (150 mM NaCl and 5 mM acetic acid in water). Cells were fixed for 10 min with 4% PFA in PBS, permeabilized for 20 min with blocking buffer (0.05% saponin, 0.5% BSA, 50 mM NH₄Cl in PBS), and incubated for 1 h at 25 °C with rabbit anti-GST^16^ and mouse anti-EEA1 antibodies in blocking buffer. After three PBS washes, the cells were incubated for 45 min at 25 °C with Alexa Fluor 568–conjugated anti-rabbit and Alexa Fluor 488–conjugated anti-mouse secondary antibodies (1:400) and DAPI (1:1000). At least five fields per well were acquired using an Opera high-content confocal system (PerkinElmer). A dedicated Harmony/Acapella script segmented EEA1-positive early endosomes, quantified the integrated GST–RAP fluorescence within these structures and reported the integrated intensity of the GST–RAP signal in the endosome. These values were then normalized per plate using a linear rescue-scale transformation, where the normalized scores and Z’ scores were calculated as described above for the primary assay using M6PR. The results of the RAP internalization assay, Z’, were 0.67, indicating that this is a reliable assay suitable for high-content screening.^4^ The untreated CTRL population corresponded to a z score of 3.7. Compounds were then ordered from highest to lowest z scores and plotted to visualize the distribution of activity and prioritize the strongest rescuing compounds.

### Immunofluorescence staining of cell lines

Cells were grown on 13 mm glass coverslips and left untreated or treated as indicated. The cells were fixed with 4% paraformaldehyde for 10 min at room temperature and then were washed three times in 1× PBS. The cells were permeabilized and blocked in blocking buffer (BB: 0.05% (w/v) saponin, 0.5% (w/v) BSA, 50 mM NH₄Cl, and 0.02% NaN₃ in PBS) for 45 min, followed by a 2 h incubation with primary antibodies in blocking buffer. The cells were then washed with PBS and incubated with Alexa Fluor–conjugated secondary antibodies (1:400) and DAPI (1:1000) diluted in blocking buffer. After immunostaining, the cells were washed twice in PBS and once in distilled water to remove salts. Coverslips were mounted on glass microscope slides with Mowiol.

### PI(4,5)P2 immunofluorescence staining

PI(4,5)P2 staining was performed with a modified version of a published protocol.^79^

For endosomal PI(4,5)P2 immunolabeling, cells (60–70% confluence) cultured in 500 µL of medium were fixed by adding 500 µL of 4% PFA in PBS to a final concentration of 2% and incubating for 15 min at room temperature, followed by quenching with three rinses in PBS containing 50 mM NH₄Cl. The cells were permeabilized for 7 min by the addition of 20 µM digitonin in Buffer A (20 mM Na-PIPES, 137 mM NaCl, 2.7 mM KCl, pH 6.8). Digitonin was removed by three rinses in Buffer A, and the cells were blocked for 45 min with Buffer A supplemented with 5% (v/v) goat serum and 50 mM NH₄Cl. Cells were incubated with primary antibodies in blocking buffer (Buffer A supplemented with 5% (v/v) goat serum) at room temperature for 1 h, followed by three rinses in Buffer A. Secondary antibodies were applied in Buffer A supplemented with 5% goat serum at room temperature for 1 h, followed by three rinses in Buffer A. Postfixation was performed for 5 min in 2% PFA at room temperature, followed by quenching with one wash in PBS containing 50 mM NH₄Cl and one wash in sterile water. Coverslips were mounted on glass microscope slides with Mowiol.

For plasma membrane PI(4,5)P2 immunolabeling, the cells were fixed with 4% PFA for 15 min and then rinsed three times with PBS containing 50 mM NH₄Cl. Slides were placed on ice for 2–5 min; all subsequent steps were performed on ice. The cells were blocked and permeabilized for 45 min with Buffer A supplemented with 5% (v/v) goat serum, 50 mM NH₄Cl, and 0.5% saponin. The cells were incubated with primary antibodies in Buffer A supplemented with 5% (v/v) goat serum and 0.1% saponin for 1 h. The cells were then rinsed twice with Buffer A and incubated with secondary antibodies in Buffer A supplemented with 5% (v/v) goat serum and 0.1% saponin for 45 min. The cells were subsequently rinsed three times with Buffer A and postfixed for 10 min with 2% PFA in PBS. The cells were returned to room temperature for 5 min, after which the PFA was removed, and the samples were rinsed three times with PBS containing 50 mM NH₄Cl. The cells were rinsed once with distilled water and mounted as described above.

### Confocal fluorescence microscopy, image processing, and colocalization analysis

HeLa cells, HK-2 cells and PTCs were grown to subconfluence on glass coverslips and processed for immunofluorescence microscopy and quantitative image analysis as follows. The experiments were repeated at least three times, and representative images are shown. Colocalization (EEA1-RAP-GST, EEA1-actin (phalloidin), EEA1-LRP2, EEA1-PI(4,5)P2) was calculated by acquiring confocal sections from ~50–100 cells per experimental condition per experiment. Confocal images (average resolution 2048 × 2048) of at least 50–100 cells per condition were acquired at the same laser power and photomultiplier gain. The images were processed using ImageJ. Single channels were converted into 16-bit grayscale images (cell lines) or 12-bit grayscale images (organoids) and thresholded to subtract background. The ImageJ “Analyze Particles” plugin was used to identify and count the total number of structures (area > 0.05 µm²) in channel 1 (EEA1). These structures were used to construct a mask overlapping with the other markers (RAP–GST, phalloidin, LRP2, and PI(4,5)P2). The fluorescence intensity of the indicated markers was measured in the overlapping structures and normalized by dividing these intensities by those of the structures in channel 1 (EEA1). This normalization accounts for possible immunostaining heterogeneity within the sample.^80^ For conventional confocal microscopy, a confocal laser microscope (Zeiss LSM 800; Carl Zeiss) with a 63 × 1.4 NA oil-immersion objective was used.

### Differentiation of kidney organoids from human iPSCs

Kidney organoids were generated from human iPSCs using an optimized version of a large-scale differentiation protocol,^40,41^ with minor modifications to standardize them across lines. For each differentiation, one 100-mm iPSC culture dish at 60–80% confluence was used to seed a single 6-well plate. Plates were treated with Anti-Adherence Rinsing Solution by adding 2 mL per well, incubating 15–20 min at room temperature, and replacing with 2 mL per well complete Stage I medium (TeSR-E5 supplemented with 0.25% PVA, 1× penicillin‒streptomycin, 0.1× ITS-E, 1× chemically defined lipid and 2.5 µg/mL Plasmocin). On day 0, the iPSC colonies at 60–80% confluence were washed twice with DPBS, incubated with 2 mL of dispase for 6 min at 37 °C, and then washed three times with DPBS to remove the enzyme. Colonies were gently scraped and triturated with a 5 mL stripette and transferred into Stage I medium supplemented with 8 µM CHIR99021, 3.3 µM ROCK inhibitor Y-27632, and 0.1 mM β-mercaptoethanol. A final volume of 3 mL per well was added to Anti-Adherence–treated 6-well plates, which were subsequently cultured on an orbital shaker inside a humidified incubator at 37 °C and 5% CO₂ (orbital 30 rpm; reciprocal 330°; vibration 5°; 2-s vibration cycle). After two days, the embryoid bodies (EBs) were allowed to settle, and the medium was reduced to ~1 mL per well and replaced with 2 mL of fresh Stage I medium containing 8 µM CHIR99021 only (no Y-27632 or β-mercaptoethanol). The following day, the EBs were collected and washed twice in low-glucose DMEM (Gibco, 31885023), and the 6-well plate was subsequently washed twice with low-glucose DMEM. The wells were then filled with 2 mL of Stage II medium (DMEM low glucose supplemented with 10% KOSR, 0.25% PVA, 1× penicillin‒streptomycin, 1× MEM-NEAA, 1× GlutaMAX, 1× HEPES, and 2.5 µg/mL Plasmocin). EBs were passed sequentially through 200 µm and 300–400 µm strainers to remove aggregates and out-of-range sizes. Size-selected EBs were resuspended in 6 mL of Stage II medium and returned to the 6-well plate at 1 mL per well (a total of 3 mL per well of Stage II medium); 2 mL of medium was removed and replaced with fresh Stage II medium every other day. Tubulogenesis typically occurred on days 7–9, with optimal maturation occurring on day 15. Organoids outside the target size range ( < 200 µm or >500 µm) were removed by sieving to maintain uniformity.

Morphological and gene expression analyses (Supplementary Fig. 2) confirmed the differentiation time course reported in refs. ^7^^,^^8^ with peak expression of differentiation markers on day 17 and only slightly lower levels at later time points that remained significantly higher than those in undifferentiated iPSCs up to day 21. The expression of differentiation markers was assessed by qRT‒PCR using the following primers: WT1 (FW: GTGACTTCAAGGACTGTGAACG and REV: CGGGAGAACTTTCGCTGACAA), NPHS1 (FW: AGTGTGGCTAAGGGATTACCC and REV: TCACCGTGAATGTTCTGTTCC), LRP2 (FW: AAATTGAGCACAGCACCTTTGA and REV: TCTGCTTTCCTGACTCGAATAATG), GATA3 (FW: GCCCCTCATTAAGCCCAAG and REV: TTGTGGTGGTCTGACAGTTCG) and HPRT (FW: CATTATGCTGAGGATTTGGAAAGG and REV: CTTGAGCACACAGAGGGCTACA). Kidney organoids were considered fully differentiated by day 14^40,41^ and were manually separated from undifferentiated spheroids. Treatment with IB-MECA (10 μM for 7 days) was therefore initiated on day 14 to avoid confounding effects on organoid differentiation. Organoids were harvested and analyzed on day 21 by immunofluorescence, endocytosis assays (BSA/Dextran uptake), and single-cell RNA sequencing.

### Immunofluorescence staining of kidney organoids

Organoids were fixed at the end of differentiation with 4% paraformaldehyde (PFA) in 1× DPBS for 45 min on an orbital shaker. The samples were subsequently washed three times in 1× DPBS and permeabilized/blocked for 4 h at room temperature in blocking buffer (6% donkey serum and 1% Triton X-100 in 1× Tris-buffered saline). Primary antibodies (LRP2/megalin; 1:200 IF) were applied overnight at 4 °C on an orbital shaker. After three washes in 1× DPBS, the sections were incubated with Alexa Fluor–conjugated secondary antibodies (Life Technologies; 1:200) for 4 h in blocking buffer at room temperature on an orbital shaker. Organoids were washed three times in 1× DPBS, mounted in Mowiol using a 9 mm-diameter, 0.12 mm-deep spacer, and imaged on an Axioscan microscope (Nikon) with a 60X, 1.4NA oil-immersion objective. Images were saved in .nd2 format and analyzed by Fiji.

### Immunofluorescence staining of kidney organoid sections

Organoids were fixed with 4% PFA (paraformaldehyde in DPBS) for 45 min, incubated overnight in 30% sucrose, embedded in Tissue-Tek OCT (optimal cutting temperature) compound (Electron Microscopy Sciences, Hatfield, USA), and sectioned at 7 µm. Kidney organoid sections were then permeabilized and blocked for 4 h in BB-kidney organoid buffer (6% donkey serum and 1% Triton X-100 in 1× Tris-buffered saline). Organoids were stained overnight at 4 °C with primary antibodies against A3AR at a 1:75 dilution and then were washed three times with 1× DPBS and stained with secondary antibodies (Alexa Fluor, Life Technologies) and LTL-FITC, both at a 1:200 dilution, for 1 h in BB-Kidney organoid buffer at room temperature. Kidney organoid sections were then washed three times in DPBS at room temperature and mounted with Mowiol using coverslips. Slides were imaged using an Axioscan microscope (Nikon) with a 60X, 1.4NA oil-immersion objective. Images were saved in .nd2 format and analyzed by Fiji.

### Endocytic assays: albumin and dextran uptake in kidney organoids

A total of 10 human kidney organoids on day 20 of differentiation were transferred to 48-well plates pretreated with anti-adhesion solution and maintained under Stage II conditions. Stage II medium without KOSR (KnockOut™ Serum Replacement, Gibco) was supplemented with BSA–Alexa Fluor 555 (final concentration of 50 µg/mL) and dextran–Alexa Fluor 647 (final concentration of 300 µg/mL) and incubated for 16 h at 37 °C. Organoids were then washed three times in DPBS, fixed in 4% PFA for 45 min, and immunostained as described above.

### Codetection of RNA (RNA Scope) and protein in human renal organoids

Hs-ADORA3 RNA was detected using a predesigned RNAscope probe (Advanced Cell Diagnostics, USA) as previously described in.^81^ RNA in situ hybridization (ISH) was performed with an RNAscope Multiplex Fluorescent Detection Reagents v2 kit (Cat. No. 323110; Advanced Cell Diagnostics). OCT-embedded organoid sections were postfixed, dehydrated, and subjected to heat-induced target retrieval at 95–100 °C using a customized protocol. The sections were then incubated with RNAscope Pretreatment Reagents (Cat. No. 323770; Advanced Cell Diagnostics) at 40 °C. The Hs-ADORA3 probe was hybridized to the tissue sections, and the signal was amplified according to the manufacturer’s protocol and visualized using a TSA Vivid Fluorophore 570 (Cat. No. 323272; Advanced Cell Diagnostics). The sections were subsequently incubated overnight at 4 °C with a rabbit polyclonal anti‑LRP2/Megalin antibody (1:200). After being washed, the sections were incubated for 1 h at room temperature with an Alexa Fluor 488‑conjugated donkey anti‑rabbit IgG secondary antibody (1:500; Molecular Probes, Eugene, OR, USA). The nuclei were counterstained with DAPI, and the sections were mounted using GEL/MOUNT (Biomeda, Foster City, CA, USA). Imaging was performed on an Axioscan microscope (Nikon) equipped with a 60× and 1.4 NA oil‑immersion objective. Images were saved in .nd2 format and analyzed with Fiji. Proximal tubule regions were manually delineated on the basis of LRP2/Megalin staining, and a constant threshold was applied across all the samples to detect the ADORA3 mRNA signal. The number of ADORA3‑positive puncta within the proximal tubule region was quantified and normalized to the corresponding area, yielding values expressed as puncta per µm² of the proximal tubule area. To quantify the signal outside the proximal tubule compartment, the previously defined proximal tubule regions were excluded from the total organoid area; ADORA3‑positive spots in the remaining area were then measured and normalized to the non‑proximal tubule area, yielding values expressed as spots per µm² of non‑proximal tubule area. High‑magnification images were acquired using a ZEISS LSM 800 confocal microscope with a 63× and 1.4 NA oil‑immersion objective.

### Single-cell RNA-seq sample preparation and analysis

Up to five organoids were pooled per sample and washed five times with 1× DPBS. Organoids were incubated with 1 mL of Accutase for 45–60 min at 37 °C with gentle mixing every 10–15 min, followed by mechanical trituration using a 25 G needle and syringe to obtain a single-cell suspension. The reaction was quenched with an equal volume of 1× DPBS. The cells were counted (Bio-Rad automated counter), pelleted (300 × g, 4 min, room temperature), resuspended in 2 mL of 1× DPBS supplemented with 10% KOSR, and filtered through a 100 µm strainer. Viability (Trypan blue) was 80–85%. Single-cell suspensions were immediately transferred to NEGEDIA S.R.L. for library preparation and sequencing. The cell suspension was loaded onto a Chromium Single-Cell G Chip Kit (10x Genomics) and processed with the Chromium Single-Cell Controller (10x Genomics) to generate single-cell gel bead emulsions, following the manufacturer’s guidelines. cDNA and final libraries were constructed using the Single-cell 3′ Library and Gel Bead Kit V4 (10x Genomics). The quality of the cDNA was checked with High-Sensitivity D5000 ScreenTape using the Agilent 4200 TapeStation system (Agilent Technologies), while the final libraries were evaluated with High-Sensitivity DNA D1000 ScreenTape (Agilent Technologies). The libraries were sequenced on a NovaSeq 6000 platform (Illumina) according to the manufacturer’s instructions.

For data processing, cluster annotation, and enrichment analysis, Raw FASTQ files were processed with Cell Ranger v9.0.1 to generate gene–barcode UMI count matrices using the 10x Genomics GRCh38 reference “refdata-gex-GRCh38-2024-A”. Filtered feature–barcode matrices were used for downstream analyses. Downstream analyses were performed in Python using Scanpy v1.11.5.^82^ Cells were retained if they met all of the following criteria: mitochondrial fraction < 20%, > 1000 detected genes, and log1p(total counts) > 8 (approximately > 3000 total counts). Genes were retained if they had ≥ 1000 total counts across the dataset, resulting in 16,259 genes for downstream analyses. The expression counts were normalized per cell to a fixed library size (10,000 counts per cell) and log-transformed (log1p). Dimensionality reduction and graph-based clustering were performed in Scanpy; UMAP embeddings^83^ were used for visualization. A k-nearest-neighbor graph was constructed using 15 neighbors, and the first 21 principal components and clusters were identified with the Leiden algorithm at a resolution of 0.2.^84^ The cluster marker genes used for cell type identification were ranked using Scanpy rank_genes_groups and used as inputs for automated annotation with CyteType.^85^ Labels were subsequently manually validated by inspecting canonical marker expression across clusters using Scanpy visualizations. Differential expression was performed within each annotated cell type across predefined condition comparisons using the Wilcoxon rank-sum test as implemented in Scanpy.^82^ Genes were considered differentially expressed if they met both criteria: FDR-adjusted *p* value < 0.05 and |log fold change | > 0.5. Functional enrichment analysis was performed on DEG lists using g:Profiler via its Python interface (v1.0.0) with default settings.^86^ For OCRL, PIP5K1A, PIP5K1B, PI4KB, and INPP5B in our dataset and the “GSE234788” dataset,^44^ the normalized mean expression is shown for each gene and cell type. The expression values were calculated from the raw counts by normalizing each gene count to the total per-cell library size and applying a log1p transformation: log1p(raw count/total counts per cell × 10,000). For GPCR analysis, GPCR expression levels were calculated on the basis of their pseudobulk logCPM expression across renal cell types and conditions. Raw counts were aggregated within each condition–cell type group, normalized by the total pseudobulk library size, and log1p(CPM) transformed.

### Proximal tubule cell isolation from kidney organoids

The protocol for isolating proximal tubule cells from kidney organoids was adapted from ref. ^16^. On day 21 of differentiation, seventy kidney organoids were washed twice with 1× DPBS and incubated with 1 mL of Accutase in a 15-mL tube for 40 min at room temperature on a shaker (600 rpm). Every 20 min, the organoids were mechanically dissociated by pipetting 20 times through a 25 G needle. The resulting cell suspension was diluted with 2 mL of 1× DPBS and centrifuged at 300 × g for 5 min at 4 °C. The supernatant was discarded, and the pellet was resuspended in 500 µL of 1× DPBS containing LTL‑FITC (Lotus tetragonolobus lectin, fluorescein conjugate) diluted 1:50. The samples were incubated on ice for 15 min and centrifuged again at 300 × g for 5 min at 4 °C, after which the supernatant was removed. Cells were resuspended in 3 mL of 1× DPBS, passed through a 35-µm cell strainer to obtain a single-cell suspension, and subsequently sorted on the basis of FITC fluorescence, taking advantage of the specific binding of LTL to proximal tubule cells.

LFL‑FITC‑positive and LTL‑FITC‑negative fractions were subjected to RNA extraction and qPCR analysis of ADORA3 and LRP2 expression, which were normalized to the expression of the housekeeping gene GAPDH. The primers used were as follows: GAPDH (FW: ACCAAATCCGTTGACTCCGAC, REV: CTCCTGTTCGACAGTCAGCC), LRP2 (FW: AAATTGAGCACAGCACCTTTGA, REV: TCTGCTTTCCTGACTCGAATAATG), and ADORA3 (FW: CACCAGAAAAGGATACAAGAGGGT, REV: ACATGGGGGTCAATCCCAC).

### Microplate reader-based Ca²⁺ assay

Experiments were performed using an automated microplate reader (FLUOstar Omega, BMG LABTECH) equipped with syringe pumps and optical excitation (500 nm) and emission (535 nm) filters. HK-2 cells, either mock-treated or transfected with siRNA targeting OCRL, were cultured for 72 h in 6-well plates and then harvested and plated at 15,000 cells/well in 96-well microplates (Corning 3603). Twenty-four hours later, the cells were washed twice with PBS (120 µL per wash) and loaded for 1 h at 37 °C with 5 µM Fluo-4/AM (Thermo Fisher Scientific) in PBS containing 10 mM glucose, 0.5 mM sulfinpyrazone, and 1% fetal bovine serum. After loading, the cells were washed twice with PBS–glucose–sulfinpyrazone (120 µL per wash) and incubated for 10 min at 37 °C with 60 µL of the same solution. Each well was assayed individually. Fluo-4 fluorescence was detected every 200 ms for 16 s. After 2 s of recording, 165 µL of PBS solution (plus 10 mM glucose and 0.5 mM sulfinpyrazone) containing a Ca²⁺-elevating agent was added: UTP (5 µM) or ionomycin (iono; 1 µM), either alone or in combination with the PLC inhibitor U73122 (10 µM). Fluo-4 fluorescence values were processed by background subtraction and normalization to the initial value measured before stimulus addition.

### Mouse models and IB-MECA treatment

Experiments were conducted on age- and sex-matched *Ocrl*^*Y/−*^; *Inpp5b* ^− /−^ mouse littermates harboring BAC-*INPP5B* expression (129S/SvEv × 129S6/SvEvTac × FVB/N × C57BL/6 background).^45^ The mice were housed under temperature- and humidity-controlled conditions with 12 h light/12 h dark cycles and free access to an appropriate standard diet and water. Eight-week-old mice were fed a control diet (#4RF21 Mice and Rats; Mucedola; *n* = 15 mice) or IB-MECA-containing chow (#4RF21 with 0.75 mg/kg IB-MECA; Mucedola; *n* = 15 mice) for 5 months. Urine samples were collected the day before the beginning of treatment and then every month. The amount of IB-MECA added to chow was set on the basis of measured daily chow consumption ( ~ 3 g/day per mouse), corresponding to a daily intake of ~100 µg/kg IB-MECA.

### Assessment of urinary and plasma biochemical parameters

The mice were housed in metabolic cages for 24 h with free access to food and water. Urine was collected, and body weight, water intake, and urine output were recorded. Urinary Clara cell protein (CC16) concentrations were measured in triplicate by enzyme‑linked immunosorbent assay (ELISA). Urinary CC16 levels were normalized to urinary creatinine levels, as determined by the Jaffe method,^18^ and values were further adjusted for body weight.

Urinary creatinine, Mg²⁺, Ca²⁺, and phosphate were quantified using a BT1500 chemistry analyzer (Biotecnica Instruments, Italy). Urine osmolality was measured with an Osmo Touch analyzer (Astori). Plasma biochemical parameters, including blood urea nitrogen (BUN), creatinine, alanine aminotransferase (ALT), and aspartate aminotransferase (AST), were determined using the VitroVet clinical chemistry system (Scil Animal Care Company) according to the manufacturer’s instructions. All analyses were performed with the corresponding VitroVet reagent slides/cartridges, calibrators, and quality‑control materials for each analyte. The samples were analyzed undiluted when the values fell within the analytical range; measurements outside the linear range were repeated after appropriate dilution, following the manufacturer’s recommendations.

### In vivo endocytosis assays

The proximal tubule endocytic capacity of untreated or IB-MECA-treated *Ocrl*^*Y/−*^ mice was examined by measuring β-lactoglobulin uptake.^22^ β-lactoglobulin was tagged with Alexa Fluor 555 in accordance with the manufacturer’s instructions. Thirty minutes after retro-orbital intravenous injection of AF555–β-lactoglobulin (1 mg/kg body weight), the mice were anesthetized (sublethal doses of ketamine and medetomidine), and the kidneys were harvested after perfusion with sterile PBS. Kidneys were fixed with 4% PFA in PBS for 8 h, incubated for 5 h in 20% sucrose in PBS and then for 16 h in 30% sucrose, and embedded in cryogenic Tissue-Tek OCT compound. Embedded tissues were cryosectioned at 10 µm.

### Immunofluorescence staining of frozen tissue sections and quantitative analysis of proximal tubule endocytosis

Sections (10 µm) were permeabilized with 0.2% Triton X-100 in PBS for 30 min and incubated overnight in a humidified chamber with LTL-FITC (1:200) and DAPI (1:1000) diluted in permeabilization buffer. After immunostaining, tissue sections were washed twice in PBS and once in sterile water, mounted with Mowiol, and analyzed by laser scanning confocal microscopy. To quantify β-lactoglobulin internalization, the integrated density of the signal within each LTL-positive tubule was measured, and internalization is expressed as the ratio of internal fluorescence to the tubule area.

### TPFE-SHG imaging and morphometric analysis

Intravital two-photon microscopy was used to assess type I collagen accumulation in kidney tissue. Imaging was performed on an upright Ultima Investigator microscope (Bruker, MS, USA) equipped with a 20× XLUMPlanFL water‑immersion objective (NA 1.41; Olympus, Japan). The Ti–sapphire laser (Mai Tai® DeepSee™, Spectra‑Physics, USA) was tuned to an excitation wavelength of 900 nm. Second-harmonic generation (SHG) was used to visualize the intrinsic signal of type I collagen in unstained 4‑µm‑thick kidney cryosections. Two-photon excited fluorescence (TPEF) autofluorescence was used to image the structural organization of the kidney cortex. The microscope was controlled using Prairie View software. For each condition, 10 images were acquired from 4 *Ocrl*^*Y/−*^ untreated mice, 4 IB‑MECA‑treated *Ocrl*^*Y/−*^ mice, and 2 *Ocrl*^*Y/+*^*(WT)* mice. Images were collected at 512 × 512 pixels with a pixel dwell time of 3.2 µs. Fibrosis was quantified in Fiji (version 2.9.0/1.53t) as the percentage of tissue area occupied by type I collagen, which was calculated as the ratio of the collagen‑positive area to the total kidney parenchymal area.

### Isolation of β-lactoglobulin-positive and -negative kidney cells for RNA sequencing

WT and *Ocrl*^*Y/−*^ mice were injected intravenously (retroorbital administration) with Alexa Fluor 555–labeled β-lactoglobulin (AF555–β-lactoglobulin; 1 mg/kg body weight) and sacrificed 30 min post-injection. Kidneys were harvested, and the cortex was isolated by manual dissection. Cortical tissue was cut into ~1 mm² pieces and enzymatically dissociated using collagenase IV to generate a single-cell suspension. The cells were subsequently washed and resuspended in PBS supplemented with 1% FBS, after which fluorescence-activated cell sorting (FACS) was performed on the basis of AF555–β-lactoglobulin fluorescence to separate β-lactoglobulin–positive (BLG⁺) and β-lactoglobulin–negative (BLG⁻) populations. Sorted BLG⁺ and BLG⁻ cells were collected, processed for RNA extraction, and subjected to bulk RNA sequencing. Total RNA was quantified using a Qubit 4.0 fluorometric assay (Thermo Fisher Scientific). Libraries were prepared from 125 ng of total RNA using the NEGEDIA Digital mRNA-seq research grade sequencing service v2.0 (Next Generation Diagnostic SRL), which included library preparation, quality assessment, and sequencing on a NovaSeq 6000 sequencing system using a single-end, 100-cycle strategy (Illumina Inc.). The raw data were analyzed by the Next Generation Diagnostic SRL proprietary NEGEDIA Digital mRNA-seq pipeline (v2.0), which involves a step involving quality filtering and trimming, alignment to the reference genome, and counting by gene. The raw expression data were normalized, analyzed by the NEGEDIA degsanalysis pipeline (v1.2.0) and visualized in a proprietary report (v1.0).^87–90^

## Supplementary information

Supplementary Materials

Supplementary Data 1

Supplementary Data 2

Supplementary Data 3

Supplementary Data 4

Supplementary Data 5

## Data Availability

Single-cell RNA-seq data supporting Fig. 2e–h and Supplementary Fig. 4 have been deposited in the ArrayExpress collection within BioStudies under accession number E-MTAB-16673 and are available at https://www.ebi.ac.uk/biostudies/ArrayExpress/studies/E-MTAB-16673?key=dc46b3f6-300c-4ba5-96cf-80ebc01b68e7. Additional datasets generated and analyzed in this study are provided as Supplementary data files. These include differential gene expression analyses of untreated and IB-MECA-treated WT and OCRL-KO kidney organoids (Supplementary Data 1), Gene Ontology Cellular Component (GO:CC) analyses of the single-cell RNA-seq dataset (Supplementary Data 2, 3), RNA-seq analysis of mouse proximal tubule cells (Supplementary Data 4), and analysis of GPCR expression in WT and OCRL-KO kidney cell clusters from kidney organoids (Supplementary Data 5).

## Code availability

The code used to perform the single-cell RNA sequencing (scRNA-seq) analysis is available in the GitHub repository (https://github.com/bioinformatics-tigem/scRNAseq-OCRL-KO-kidney-organoids).
