# Supplementary material for: An actionable GPCR restores phosphoinositide homeostasis and kidney function in Lowe syndrome
Source: Signal Transduct Target Ther. 2026 Nov 4;11:482. doi: 10.1038/s41392-026-03054-z (PMC13731648; doi:10.1038/s41392-026-03054-z)
Supplement: Supplementary file 1 [file 41392_2026_3054_MOESM1_ESM.docx]

Supplementary Materials for

**An Actionable GPCR to Restore Phosphoinositide Homeostasis and Renal Function in Lowe Syndrome**

Leopoldo Staiano^1,2^*, Marialuisa Testa^1^, Mariella Vicinanza^3^, Francesca Cervellini^1,4^, Laura Giaquinto^1,5^, Lorenzo Patanella^1^, Giuseppe Di Tullio^1^, Michele Santoro^1^, Annamaria Carissimo^6^, Alessandro Giordano^1^, Michele Genovese^1^, Luis Juan Vicente Galietta^1,7^, Anna Iervolino^8^, Antonio Miele^8^, Diego Luis Medina^1,7^, Francesco Trepiccione^8,9^, Francesco Emma^10^, Olivier Devuyst^11,12^, Robert Nussbaum^13^, and Maria Antonietta De Matteis^1,5^*

*Correspondence to [l.staiano@tigem.it](mailto:l.staiano@tigem.it) or [dematteis@tigem.it](mailto:dematteis@tigem.it)

**This PDF file includes:**

Supplementary Figures 1 to 8

Supplementary Tables 1 to 3

Captions for Supplementary Data 1, 2, 3, 4 and 5

Supplementary References

**Other Supplementary Materials for this manuscript include 5 excel files indicated as**

Supplementary Data 1 to 5.

Supplementary Data 1. Differential Gene Expression analysis in kidney cell clusters in untreated and treated WT and untreated and treated OCRL-KO kidney organoids.

Supplementary Data 2. GSEA of GO:Cellular Component terms in OCRL-KO vs WT kidney organoids.

Supplementary Data 3. GSEA of GO:Cellular Component terms in kidney cell clusters in OCRL-KO+IB-MECA vs OCRL-KO and in OCRL-KO+IB-MECA vs WT kidney organoids.

Supplementary Data 4. Bulk RNA-seq of isolated proximal tubule cells from WT and Lowe (*Ocrl^y/-^)* mice.

Supplementary Data **5. GPCR expression in kidney cell clusters in WT and OCRL-KO kidney organoids.**

**Supplementary Figure 1. Characterization of the inducible OCRL-KD cell line and secondary screening/validation of LOPAC hits.**

**(a)** Western blot and quantitative analysis of OCRL expression in HeLa cells stably transfected with a tetracycline-inducible shRNA against OCRL (Tet-regulated shRNA-OCRL clone). OCRL protein levels were reduced by >80% upon tetracycline treatment (+ Tet) compared with untreated cells (− Tet). Actin is shown as a loading control. **(b)** Quantitative analysis of M6PR distribution in OCRL-KD HeLa cells treated or not with the PI4KIIIβ inhibitor PIK93 (500nM). One-way Anova with Tukey’s post hoc test was used for statistical analysis and p-values are reported on the graph. **(c)** Secondary high-content RAP-GST internalization assay in OCRL-KD HK-2 cells. The graph shows the 18 compounds that rescued RAP-GST internalization to >50% of CTRL levels (0.5 on the Y-axis) in at least 2 of the 3 concentrations tested in the secondary assay. RAP-GST uptake, expressed as normalized RAP-GST integrated density per cell (CTRL set to 1,). PIK93 and Wortmannin were used as positive controls. **(d)** Quantitative analysis of RAP uptake in proximal tubule cells (PTCs) from Healthy Donors (CTRL) measured as Endosomal RAP integrated density in endosomes/cell, after treatment with 18 small molecules (at the concentration used in the primary screening, i.e. 10 μM) selected from the 113 molecules tested in (Fig. 1e). PIK-93 (500nM) was included as a positive control. IB-MECA is shown in green. The inhibitory effect of IB-MECA on RAP uptake in healthy donor PTCs seen at 10 μM was absent at 250 nM, which was the concentration used in subsequent experiments for validation and mechanistic studies. **(e)** Quantitative analysis of endosomal actin accumulation (phalloidin) on EEA1-positive endosomes in proximal tubule cells (PTCs) from Healthy Donors and LS patients treated with the 18 selected compounds. **(f)** Quantitative analysis of autophagosome number (LC3-positive puncta) in proximal tubule cells (PTCs) from Healthy Donors and LS patients treated with the 18 selected compounds and with PIK93 (500 nM) as positive control. In (d), (e) and (f), IB-MECA treatment is highlighted in green. Bars are per-cell ± SD with at least 100 cells from 3 independent biological replicates (n > 300, N = 3). Statistical analysis was performed by Kruskal–Wallis with appropriate post-hoc tests.

**Supplementary Figure 2**. **Gene expression analysis of developing kidney organoids.**

(a) qPCR analysis of lineage‑specific nephron markers in WT and OCRL‑KO iPSCs and during differentiation to kidney organoids. Expression of the podocyte markers WT1 and NPHS1, the proximal tubule marker LRP2, and the distal nephron marker GATA3 was assessed at multiple stages of the differentiation protocol, including undifferentiated iPSCs and organoids at days 17, 21, and 25. In both WT and OCRL‑KO organoids, all markers showed a robust induction during differentiation compared with the corresponding iPSC stage, consistent with progressive acquisition of nephron lineage identities. For several markers, expression peaked at day 17 and declined at later time points. However, expression levels at day 21 remained markedly higher than in iPSCs, indicating sustained presence of differentiated nephron cell populations within the organoids, as tested by single-cell RNA sequencing performed at day 21 which identified all principal nephron cell classes, together with expected extra-renal cell types (Fig. 2e). Gene expression values were normalized to HPRT1 and are presented relative to the levels detected in iPSCs. Data represent mean ± SD from three independent biological replicates.

**Supplementary Figure 3. ADORA3/A3AR is expressed in the cellular, organoid, and mouse models used to assess IB‑MECA responses.**

(a) Box plot showing RT–qPCR analysis of ADORA3 expression in WT and OCRL‑KO kidney organoids, expressed as 2^-dCt relative to GAPDH. Data are from three independent experiments. (b) Representative immunofluorescence images of WT and OCRL‑KO kidney organoids stained for A3AR and the proximal tubule marker LTL‑FITC, showing a detectable A3AR signal in proximal tubule–like structures. Scale bar, 50 μm. (c) Box plot showing RT–qPCR analysis of ADORA3 expression in primary proximal tubule cells (PTCs) derived from healthy donors and Lowe patients, expressed as 2^-dCt relative to GAPDH. Data are from four independent experiments. (d) Representative immunofluorescence images of healthy donor‑ and Lowe patient‑derived PTCs stained for A3AR together with Na⁺/K⁺‑ATPase. Scale bar, 20 μm. (e) Box plot showing RT–qPCR analysis of ADORA3 expression in control and OCRL‑KD HK-2 cells, expressed as 2^-dCt relative to GAPDH. Data are from four independent experiments. (f) Representative immunofluorescence images of HK-2 cells stained for A3AR together with Na⁺/K⁺‑ATPase, used as a plasma membrane marker. Scale bar, 20 μm. (g) Box plot showing Adora3 expression in mouse proximal tubule cells (mPTCs) isolated from *Ocrl^Y/+^* and *Ocrl^Y/−^* mice, expressed as normalized counts. Data are from n = 5 *Ocrl^Y/+^* and n = 5 *Ocrl^Y/−^* mice. The complete bulk RNA‑seq data set from isolated proximal tubule cells of WT (*Ocrl^Y/+^* ) and Lowe (*Ocrl^Y/−^*) mice is provided in Supplementary Data 4.

Supplementary Figure 4. IB-MECA treatment does not affect kidney organoid differentiation.

(a) UMAP representation of single-cell RNA-sequencing data from WT and OCRL-KO kidney organoids treated with IB-MECA. Major kidney organoid cell populations are preserved in both conditions, including proximal tubule epithelial cells, podocytes, distal nephron epithelial cells, kidney interstitial fibroblasts, immature neuronal populations, and proliferating mesenchymal cells, indicating that IB-MECA treatment does not overtly alter organoid differentiation or cellular composition. (b) Dot plots showing Gene Ontology Cellular Component (GO:CC) enrichment analysis of differentially expressed genes in OCRL-KO organoids treated with IB-MECA compared with untreated OCRL-KO organoids, and in OCRL-KO organoids treated with IB-MECA compared with untreated WT organoids. Enriched terms are shown separately for downregulated and upregulated genes across selected kidney organoid cell populations. Dot size indicates the number of genes contributing to each term, and colour indicates statistical significance, expressed as −log10 adjusted P value. The limited GO:CC enrichment observed in the OCRL-KO + IB-MECA versus OCRL-KO comparison supports the absence of major transcriptional remodeling upon treatment, while persistent differences in the OCRL-KO + IB-MECA versus WT comparison indicate that IB-MECA does not broadly normalize the OCRL-KO transcriptional state.

**Supplementary Figure 5. IB-MECA reduces endosomal actin accumulation in OCRL-deficient cells via A3 adenosine receptor.**

**(a)** Representative confocal images of CTRL and OCRL-KD HK-2 cells co-stained with phalloidin (F-actin) and anti-EEA1, showing endosomal actin accumulation in OCRL-KD cells and its reduction upon IB-MECA treatment (250 nM, 16 h). Co-treatment with the A3 adenosine receptor antagonist MRS1220 (IB-MECA 250 nM + MRS1220 250 nM, 16 h) prevents this effect, showing prominent endosomal actin staining. Scale bars, 10 µm. **(b)** Quantitative analysis of actin-positive EEA1 endosomes for the conditions in (a). Bars represent per-cell values from at least 100 cells per condition from 3 independent biological replicates (n > 300, N = 3). Statistical analysis was performed by Kruskal–Wallis with appropriate post-hoc tests, with exact p values reported on the graph. **(c)** Quantitative real-time PCR analysis of ADORA3 mRNA levels in CTRL, ADORA3-KD and OCRL/ADORA3-KD HK-2 cells. Relative expression was calculated using the 2^-ΔΔCt method, normalizing ADORA3 mRNA to the housekeeping gene (GAPDH) and expressing values relative to CTRL cells (set to 1). Bars represent mean ± SD from 3 independent biological replicates (N = 3). Statistical analysis was performed by one-way ANOVA followed by Holm–Sidak post hoc test.

**Supplementary Figure 6. IB-MECA reduces plasma membrane and endosomal PI(4,5)P_2_ in a PLC-dependent manner.**

**(a)** Quantitative analysis of translocation of the PH-PLCδ probe from the plasma membrane to the cytosol upon IB-MECA treatment in Control HK-2 cells, measured as a decrease in PH-PLCδ probe fluorescence intensity in the region close to the plasma membrane, indicative of PI(4,5)P_2_ hydrolysis. **(b)** Representative traces from control HK-2 cells loaded with the Ca^2+^-sensitive dye Fluo-4 and treated with IB-MECA (100 μM) or with IB-MECA (100 μM) and U73122 (10 μM), as indicated. IB-MECA triggers a transient increase in cytosolic Ca^2+^, which is prevented by co-administration of U73122, an inhibitor of phospholipase C (PLC), consistent with IP_3_ production downstream of PLC activation. **(c)** Representative images of CTRL HK-2 cells either untreated or treated for 16 h with IB-MECA (250 nM), U73122 (0.5 µM) or IB-MECA + U73122, co-immunolabeled for the plasma membrane pool of PI(4,5)P_2_ and EEA1. Scale bars, 20 µm. **(d)** Quantitative analysis of plasma membrane PI(4,5)P_2_ intensity for the conditions in (c). Points are per-cell with at least 100 cells from 3 independent biological replicates (n > 300, N = 3). **(e)** Representative images of CTRL HK-2 cells under the same treatments as in (c) but co-immunolabeled for endosomal PI(4,5)P_2_ and EEA1. Scale bars, 20 µm. **(f)** Quantitative analysis of endosomal PI(4,5)P_2_ intensity (colocalizing with EEA1) for the conditions in (e). The barplot was generated with per-endosome/per-cell values with at least 100 cells from 3 independent biological replicates (n > 300, N = 3). Statistical analysis was performed by Kruskal–Wallis with Dunn’s multiple comparison post-hoc test with p values shown on the plot.

**Supplementary Figure 7. UTP stimulation reduces endosomal PI(4,5)P_2_ in OCRL-deficient cells by activating PLC.**

Stimulation of other Gq/PLC-coupled GPCRs in OCRL-KD HK-2 cells, including purinergic P2Y receptors, induces PLC-dependent cytosolic Ca^2+^ transients, as measured by Fluo-4 fluorescence. **(a)** Representative traces from control and OCRL-KD HK-2 cells loaded with the Ca^2+^-sensitive dye Fluo-4 and treated with UTP (5 μM) alone or in combination with U73122 (10 μM). UTP triggers a transient increase in cytosolic Ca^2+^, prevented by coadministration of U73122, consistent with IP_3_ production downstream of PLC activation. **(b)** Representative traces from control and OCRL-KD HK-2 cells loaded with the Ca^2+^-sensitive dye Fluo-4 and treated with Ionomycin (1 μM) alone or in combination with U73122 (10 μM). PLC inhibition by U73122 does not prevent Ca^2+^ release induced by Ionomycin. **(c**) Representative images of CTRL and OCRL-KD HK-2 cells either untreated or treated with UTP (10 µM), co-immunolabeled for endosomal PI(4,5)P_2_ and EEA1. Scale bars, 20 µm. **(d)** Quantitative analysis of endosomal PI(4,5)P_2_ intensity (colocalizing with EEA1) for the conditions in (c). Bar plots are generated from per-cell values with at least 100 cells from 3 independent biological replicates (n > 300, N = 3). Statistical analysis was performed by Kruskal–Wallis with Dunn’s multiple comparison post-hoc test, with p values reported on the graph.

**Supplementary Figure 8. Uncropped western blot images.**

| Compound Name | Normalized cell count (perinuclear continuous MPR; OCRL-KD=0, CTRL=1) |
| --- | --- |
| Sanguinarine chloride | 1,68 |
| AC-93253 iodide | 1,35 |
| 2-Phenylaminoadenosine | 1,23 |
| Carboplatin | 1,19 |
| Cantharidin | 1,12 |
| IC 261 | 1,11 |
| NNC 55-0396 | 1,06 |
| Bromoacetyl alprenolol menthane | 1,00 |
| Cantharidic Acid | 0,99 |
| Cephalosporin C zinc salt | 0,99 |
| Propafenone hydrochloride | 0,99 |
| Wortmannin from Penicillium funiculosum | 0,99 |
| Piroxicam | 0,94 |
| Fusidic acid sodium | 0,94 |
| Phosphomycin disodium | 0,91 |
| Phenelzine sulfate | 0,91 |
| N-Methyl-beta-carboline-3-carboxamide | 0,90 |
| Propionylpromazine hydrochloride | 0,89 |
| Praziquantel | 0,89 |
| Trequinsin hydrochloride | 0,88 |
| SKF 86466 | 0,88 |
| Aurothioglucose | 0,88 |
| Salmeterol xinafoate | 0,87 |
| Iodoacetamide | 0,86 |
| Protoporphyrin IX disodium | 0,85 |
| Reactive Blue 2 | 0,85 |
| Betamethasone | 0,85 |
| Tetraethylammonium chloride | 0,85 |
| Orphenadrine hydrochloride | 0,84 |
| Phorbol 12-myristate 13-acetate | 0,82 |
| Protriptyline hydrochloride | 0,82 |
| Budesonide | 0,82 |
| Phentolamine mesylate | 0,81 |
| Corticosterone | 0,80 |
| SU 4312 | 0,80 |
| LY-367,265 | 0,79 |
| Thapsigargin | 0,79 |
| Brefeldin A from Penicillium brefeldianum | 0,79 |
| Felbamate | 0,78 |
| Spiperone hydrochloride | 0,78 |
| Ketoprofen | 0,77 |
| Lidocaine N-methyl hydrochloride | 0,77 |
| Ketotifen fumarate | 0,76 |
| Endothall | 0,76 |
| Loperamide hydrochloride | 0,76 |
| Lamotrigine | 0,76 |
| L-703,606 oxalate | 0,75 |
| Methyl beta-carboline-3-carboxylate | 0,75 |
| Thio-NADP sodium | 0,75 |
| Ivermectin | 0,75 |
| loxoprofen | 0,75 |
| ARL 67156 trisodium salt | 0,75 |
| (±)-Sotalol hydrochloride | 0,74 |
| (ｱ)-Sotalol hydrochloride | 0,74 |
| Amoxapine | 0,73 |
| Oxotremorine sesquifumarate salt | 0,73 |
| Nortriptyline hydrochloride | 0,73 |
| Ethosuximide | 0,73 |
| Pheniramine maleate | 0,73 |
| 8-Bromo-cGMP sodium | 0,73 |
| SB-366791 | 0,73 |
| S(+)-Raclopride L-tartrate | 0,73 |
| Chlorambucil | 0,72 |
| Tyrphostin AG 494 | 0,72 |
| Loratadine | 0,71 |
| SR 57227A | 0,71 |
| Cortisone 21-acetate | 0,71 |
| Ro 90-7501 | 0,70 |
| Caffeic Acid | 0,70 |
| Fenoterol hydrobromide | 0,69 |
| L-162,313 | 0,69 |
| Cilostazol | 0,69 |
| Sobuzoxane | 0,69 |
| S-(+)-PD 123177 trifluoroacetate salt hydrate | 0,69 |
| Tyrphostin AG 808 | 0,69 |
| K 185 | 0,69 |
| Labetalol hydrochloride | 0,68 |
| (±)-Verapamil hydrochloride | 0,68 |
| (ｱ)-Verapamil hydrochloride | 0,68 |
| Dihydroergocristine methanesulfonate | 0,68 |
| I-OMe-Tyrphostin AG 538 | 0,67 |
| U-74389G maleate | 0,67 |
| Pergolide methanesulfonate | 0,66 |
| Alinidine | 0,66 |
| Tyrphostin AG 490 | 0,66 |
| Tyrphostin AG 555 | 0,66 |
| cis-(Z)-Flupenthixol dihydrochloride | 0,66 |
| L-Buthionine-sulfoximine | 0,66 |
| SKF 83959 hydrobromide | 0,65 |
| SKF 94836 | 0,65 |
| 8-(p-Sulfophenyl)theophylline | 0,65 |
| (ｱ)-Chlorpheniramine maleate | 0,65 |
| Bromoacetylcholine bromide | 0,65 |
| L-Leucinethiol, oxidized dihydrochloride | 0,65 |
| Benzamil hydrochloride | 0,64 |
| R(+)-6-Bromo-APB hydrobromide | 0,64 |
| LY-294,002 hydrochloride | 0,64 |
| 4-Phenyl-3-furoxancarbonitrile | 0,64 |
| Ketorolac tris salt | 0,64 |
| (ｱ)-Epinephrine hydrochloride | 0,64 |
| Rutaecarpine | 0,63 |
| Spironolactone | 0,63 |
| PQ401 | 0,63 |
| R(-)-Denopamine | 0,62 |
| Moxonidine hydrochloride | 0,61 |
| LFM-A13 | 0,61 |
| Lomefloxacin hydrochloride | 0,61 |
| Spermine tetrahydrochloride | 0,61 |
| Risperidone | 0,61 |
| NCS-382 | 0,61 |
| Aminobenztropine | 0,60 |
| 5alpha-Pregnan-3alpha-ol-11,20-dione | 0,60 |
| Noscapine hydrchloride | 0,60 |
| IB-MECA | 0,60 |

Supplementary Table 1. List of the 113 compounds identified as hits in the primary high-content screen.

All hits from the primary assay are reported together with their rescue of the CI-M6PR phenotype, determined from the proportion of cells displaying a continuous perinuclear M6PR pattern. Values represent the mean rescue at the screening concentration, normalized between OCRL-KD cells, assigned a value of 0, and CTRL cells, assigned a value of 1.

Supplementary Table 2.

Summary of urinary and plasma parameters in untreated and IB-MECA–treated mice at Baseline and after 5 months of treatment (5M). Values are expressed as mean ± SD for each group (n = 10 for Ocrl^Y/+^, n = 16 for Ocrl^Y/-^ untreated, n = 15 for Ocrl^Y/-^ treated with IB-MECA). Parameters include urinary creatinine (mg/dL), urinary volume (mL), CC16 urinary excretion (µg/24 h), body weight (g), diuresis (µL/g/min), urinary (U) calcium, magnesium, and phosphate levels normalized on urinary creatinine and urine osmolarity. Additional systemic parameters measured at 5M include BUN (mg/dL), plasma creatinine (mg/dL), AST (U/L) and ALT (U/L). Between–group comparisons (Untreated vs IB-MECA at each time point) were performed using the Mann–Whitney U test. A p-value < 0,05 was considered statistically significant. ***p<0.001.

| **System** | **Dataset / reference** | **Modality** | **PT / tubule population used** | **Evidence that ADORA3 is expressed in PT / tubule epithelium** |
| --- | --- | --- | --- | --- |
| **Mouse** | **Healthy Mouse Dataset**: Wu et al., *JASN* 2019; RBK RID 14-4KBC^1^ | scRNA-seq | **PT(S1–S2)** and **PT(S3)** clusters | **Adora3 signal is detectable within PT(S1–S2) and PT(S3)** clusters. |
| **Mouse** | **Mouse kidney multimodal spatial analysis**: Xuanyuan et al., *Nat Commun* 2025^2^ | Spatial (Visium; multimodal) | **PTS1/PTS2/PTS3** in **Sham/controls** | **Adora3 is detected in proximal tubule segment**. |
| **Human PT cell lines** | **HK-2 proximal tubule epithelial cells**: Lee & Emala, *Experimental Nephrology* 2002^3^ | Immunoblot (protein) | HK-2 (**human proximal tubule** line) | Paper reports **immunoblot detection of A3AR (ADORA3)** in HK-2 cells. |
| **Human kidney** | **SHARE-seq human kidney anatomical regions**: Li et al., *Cell Metab* 2024^4^ | SHARE-seq (RNA + ATAC) | **PT and PT-state clusters** | **ADORA3 RNA signal is present in PT-labeled tubular epithelial populations**. |
| **Human kidney** | **Human kidney IHC** (normal tissue shown): Kretschmar et al., *PLOS ONE* 2016^5^ | Immunohistochemistry | **Tubular epithelium** in normal human kidney sections | **A3AR localization is reported in epithelial tubule cells** by I**HC staining** in human kidney tissue. |

**Supplementary Table 3. Evidence for ADORA3 (A3 adenosine receptor) expression in proximal tubule and tubular epithelium across healthy mouse kidney, healthy human kidney, and kidney organoid models**.

This table summarizes complementary single-cell, spatial, bulk RNA-seq, and immunodetection evidence supporting **ADORA3/Adora3** expression in **proximal tubule (PT)** or **tubular epithelial** compartments across model systems. Mouse healthy kidney scRNA-seq (Wu et al., JASN 2019; RBK RID 14-4KBC) shows detectable **Adora3** signal in PT subclusters (**PT[S1–S2], PT[S3]**). Mouse spatial multimodal profiling (Xuanyuan et al., Nat Commun 2025) shows **Adora3** detection in PT segment annotations (**PTS1–PTS3**) in sham/healthy control tissue. In human proximal tubule epithelial cell models, literature supports **ADORA3/A3AR** expression in HK-2 cells by immunoblot. Finally, human kidney SHARE-seq (Li et al., Cell Metab 2024) indicates **ADORA3** signal within PT-labeled tubular epithelial states, and immunohistochemistry evidence in human kidney tissue shows **A3AR** staining localized to tubular epithelium in normal kidney sections.

**Supplementary Data 1**. **Differential Gene Expression analysis in kidney cell clusters in untreated and IB-MECA-treated WT and OCRL-KO kidney organoids**.

Excel workbook reporting differential gene expression analyses performed across selected kidney organoid cell populations: podocytes, proximal tubule epithelial cells, distal nephron epithelial cells, and kidney interstitial fibroblasts. The workbook includes a README sheet explaining the file structure, abbreviations, row order, and color coding. Each subsequent sheet corresponds to one specific comparison within one cell population and is named according to the format Comparison_CellType.

The following comparisons are reported for each cell type: untreated OCRL-KO versus untreated WT organoids (KO_NT_vs_WT_NT), IB-MECA-treated WT versus untreated WT organoids (WT_IB_vs_WT_NT), IB-MECA-treated OCRL-KO versus untreated OCRL-KO organoids (KO_IB_vs_KO_NT), IB-MECA-treated OCRL-KO versus untreated WT organoids (KO_IB_vs_WT_NT), and IB-MECA-treated OCRL-KO versus IB-MECA-treated WT organoids (KO_IB_vs_WT_IB). Cell-type abbreviations used in the sheet names are: Podo, podocytes; Prox, proximal tubule epithelial cells; Distal, distal nephron epithelial cells; Fibro, kidney interstitial fibroblasts. Treatment abbreviations are: WT, wild type; KO, OCRL knockout; NT, non-treated; IB, IB-MECA-treated.

For each gene, the tables report the gene name, differential expression score, log fold change, nominal p value, adjusted p value, analyzed cell type, experimental condition, control condition, comparison name, and the number of cells included in the condition and control groups. Positive log fold change values indicate genes enriched in the condition listed first in the comparison, whereas negative log fold change values indicate genes enriched in the corresponding control group. Rows are ordered to facilitate interpretation: significantly upregulated genes are listed first, followed by significantly downregulated genes and then non-significant genes. Significance was defined as adjusted p value < 0.05. Color coding is used consistently across sheets: light green indicates significantly upregulated genes, light red indicates significantly downregulated genes, and light grey indicates non-significant genes.

The KO_NT_vs_WT_NT comparisons correspond to the transcriptional changes associated with OCRL loss in untreated kidney organoids and are related to the data shown in Fig. 2f. The additional comparisons allow evaluation of the transcriptional effects of IB-MECA treatment in WT and OCRL-KO organoids and assessment of the extent to which treated OCRL-KO cell populations remain transcriptionally distinct from untreated or treated WT counterparts.

**Supplementary Data 2. GSEA of GO:Cellular Component terms in OCRL-KO vs WT kidney organoids.**

Excel workbook reporting Gene Set Enrichment Analysis (GSEA) results for Gene Ontology Cellular Component (GO) terms enriched in OCRL-KO compared with WT kidney organoids, based on the cell-type-resolved scRNA-seq analysis shown in Fig. 2e and Fig. 2g. The workbook includes a README sheet describing the file content and eight result sheets corresponding to four selected kidney organoid cell populations: proximal tubule epithelial cells, kidney interstitial fibroblasts, distal nephron epithelial cells, and podocytes. For each cell population, two separate sheets are provided: one listing GO terms enriched among genes upregulated in OCRL-KO versus WT organoids and one listing GO terms enriched among genes downregulated in OCRL-KO versus WT organoids. Sheet names follow the format KO vs WT CellType Up GO_CC or KO vs WT CellType Down GO_CC, where Prox indicates proximal tubule epithelial cells, Fibro indicates kidney interstitial fibroblasts, Distal indicates distal nephron epithelial cells, and Podo indicates podocytes. Each sheet reports, for every enriched GO term, the GO database/source, GO identifier, term name, p value, significance flag, and term description. Additional columns provide enrichment and overlap metrics, including term_size, query_size, intersection_size, effective_domain_size, precision, and recall. These parameters indicate the size of the GO term, the size of the input gene set, the number of overlapping genes between the input list and the GO term, the background gene universe, and the relative strength of the overlap. The query column indicates the gene set used for the enrichment analysis, while the parents column reports the ontology hierarchy. The intersections column lists the genes contributing to each enriched term, and the evidences column reports supporting annotation information as exported from the enrichment analysis. These results were used to support the selection and visualization of representative GO categories shown in the corresponding figure panels (Fig. 2g). The README sheet is included to guide interpretation of the workbook. In addition, the workbook reports the criteria used to prioritize informative and non-redundant GO terms for visualization, including the exclusion of overly broad categories, very small gene sets, and highly overlapping or redundant ontology terms.

**Supplementary Data 3. GSEA of GO:Cellular Component terms in kidney cell clusters in OCRL-KO+IB-MECA vs OCRL-KO and in OCRL-KO+IB-MECA vs WT kidney organoids.**

Excel workbook reporting Gene Set Enrichment Analysis (GSEA) results for Gene Ontology Cellular Component (GO) terms identified in cell-type-resolved scRNA-seq comparisons involving IB-MECA-treated OCRL-KO kidney organoids. The workbook includes GO enrichment analyses for two main comparisons: OCRL-KO organoids treated with IB-MECA versus untreated OCRL-KO organoids, and OCRL-KO organoids treated with IB-MECA versus WT organoids.

The analyses are reported for four selected kidney organoid cell populations identified by scRNA-seq: proximal tubule epithelial cells, kidney interstitial fibroblasts, distal nephron epithelial cells, and podocytes. For each cell population and each comparison, two sheets are provided: one listing GO terms enriched among genes upregulated in the first condition of the comparison and one listing GO terms enriched among genes downregulated in the first condition of the comparison.

Sheet names follow the format Comparison_CellType_Up/Down_GO_CC. Cell-type abbreviations are: Prox, proximal tubule epithelial cells; Fibro, kidney interstitial fibroblasts; Distal, distal nephron epithelial cells; and Podo, podocytes. Treatment and genotype abbreviations are: WT, wild type; KO, OCRL knockout; NT, untreated; and IB, IB-MECA-treated.

Each sheet reports, for every enriched GO term, the GO database/source, GO identifier, term name/description, enrichment p value, significance flag, and set overlap metrics, including term_size, query_size, intersection_size, effective_domain_size, precision, and recall. These columns indicate the size of the GO term, the size of the input gene list, the number of genes shared between the input list and the GO term, the background gene universe used for the analysis, and the relative strength of the enrichment. The query column indicates the input gene set used for the enrichment analysis. The parents column reports the ontology hierarchy, while the intersections column lists the overlapping genes contributing to each enriched term and the evidences column reports the supporting annotation information exported from the analysis.

The workbook also includes a README/filtering summary describing the criteria used to prioritize representative and non-redundant GO terms for visualization, including the exclusion of overly broad or overly small terms and the simplification of highly overlapping ontology categories.

**Supplementary Data 4. Bulk RNA-seq of isolated proximal tubule cells from WT and Lowe (*Ocrl^y/-^)* mice.**

Excel file containing normalized and annotated bulk RNA-seq expression data from isolated proximal tubule cells collected from 5 WT and 5 Lowe mice. For each detected mRNA, the table reports gene identifiers and annotations (e.g., Symbol, GeneID, Alias, Description, Ensembl gene/transcript/peptide IDs) together with the normalized expression values for each individual sample (5 WT mice: wt724, wt727, wt749, wt750, wt752 and 5 *Ocrl-KO* mice: ko707, ko708, ko713, ko733, ko739). For WT and Ocrl-KO mice, mean expression values across replicates and mean difference in Ocrl-KO vs WT are reported. Data from this dataset were extracted and are presented in Figure S3g.

**Supplementary Data 5. GPCR expression in kidney cell clusters in WT and OCRL-KO kidney organoids.**
Excel file reporting GPCR expression levels across selected renal cell types and genotypes in WT and OCRL-KO kidney organoids. Expression values were calculated from pseudobulk logCPM profiles generated for each condition–cell type group. Raw counts were aggregated within each group, normalized to the corresponding pseudobulk library size, and reported as CPM and log1p(CPM). Data are provided for WT and OCRL-KO podocytes, proximal tubule epithelial cells, distal nephron epithelial cells, and kidney interstitial fibroblasts.

**Supplementary References**

1.	Wu, H., Kirita, Y., Donnelly, E. L. & Humphreys, B. D. Advantages of Single-Nucleus over Single-Cell RNA Sequencing of Adult Kidney: Rare Cell Types and Novel Cell States Revealed in Fibrosis. *J. Am. Soc. Nephrol.* **30**, 23–32 (2019).

2.	Xuanyuan, Q. *et al.* Multimodal spatial transcriptomic characterization of mouse kidney injury and repair. *Nat. Commun.* **16**, (2025).

3.	Lee, H. T. & Emala, C. W. Characterization of adenosine receptors in human kidney proximal tubule (HK-2) cells. *Exp. Nephrol.* **10**, 383–392 (2002).

4.	Li, H. *et al.* Transcriptomic, epigenomic, and spatial metabolomic cell profiling redefines regional human kidney anatomy. *Cell Metab.* **36**, 1105-1125.e10 (2024).

5.	Kretschmar, C. *et al.* Reduced Adenosine Uptake and Its Contribution to Signaling that Mediates Profibrotic Activation in Renal Tubular Epithelial Cells: Implication in Diabetic Nephropathy. *PLoS One* **11**, (2016).
